# Integrated analysis of FKBP1A/SLC3A2 axis in everolimus inducing ferroptosis of breast cancer and anti-proliferation of T lymphocyte

**DOI:** 10.7150/ijms.84872

**Published:** 2023-06-26

**Authors:** Zihan Chen, Rongxue Li, Min Fang, Ying Wang, Aihong Bi, Lixian Yang, Tao Song, Yucheng Li, Qiang Li, Baihua Lin, Yongshi Jia, Shi Fu, Shuiqiao Fu, Hanchu Xiong

**Affiliations:** 1 Surgical Intensive Care Unit, First Affiliated Hospital, Zhejiang University, Hangzhou, Zhejiang, 310016, China.; 2 Cancer Center, Department of Radiation Oncology, Zhejiang Provincial People's Hospital, People's Hospital of Hangzhou Medical College, Hangzhou, Zhejiang, 310014, China.

**Keywords:** FKBP1A, SLC3A2, Everolimus, Ferroptosis, Breast cancer, Th9.

## Abstract

**Background**: Solute Carrier Family 3 Member 2 (SLC3A2) is a member of the solute carrier family that plays pivotal roles in regulation of intracellular calcium levels and transports L-type amino acids. However, there are insufficient scientific researches on the prognostic and immunological roles of SLC3A2 in breast cancer (BC) and whether everolimus regulates novel SLC3A2 related molecular mechanism in the immuno-oncology context of the tumor microenvironment (TME), therefore, we see a necessity to conduct the current *in silico* and biological experimental study.

**Methods**: Using diverse online databases, we investigated the role of SLC3A2 in therapy response, clinicopathological characteristics, tumor immune infiltration, genetic alteration, methylation and single cell sequencing in BC. WB, Co-IP, cell proliferation assay, Edu staining, ROS and GSH assay and *in vivo* tumor xenograft assays were performed to verify FKBP1A/SLC3A2 axis in everolimus inducing ferroptosis of breast cancer. Co-cultures and IL-9 ELISA were performed to demonstrate the T lymphocyte function.

**Results**: We demonstrated that SLC3A2 was aberrantly expressed among various BC cohorts. Our results also suggested that SLC3A2 expression was associated with chemotherapeutic outcome in BC patients. Our results further indicated that SLC3A2 was associated with tumor infiltration of cytotoxic T cell but not other immune cells among BC TME. The alterations in SLC3A2 gene had a significant correlation to relapse free survival and contributed a significant impact on BC tumor mutational burden. Finally, SLC3A2 was illustrated to be expressed in diverse BC cellular populations at single cell level, and negatively linked to angiogenesis, inflammation and quiescence, but positively correlated with other functional phenotypes. Noteworthily, everolimus (a targeted therapy drug for BC) related protein, FK506-binding protein 1A (FKBP1A) was found to bind with SLC3A2, and negatively regulated SLC3A2 expression during the processes of everolimus inducing ferroptosis of BC cells and promoting anti-proliferation of Th9 lymphocytes.

**Conclusions**: Altogether, our study strongly implies that SLC3A2 is an immuno-oncogenic factor and FKBP1A/SLC3A2 axis would provide insights for a novel immunotherapy approach for the treatment of BC in the context of TME.

## Background

Breast cancer (BC) is a serious health problem worldwide among women aged 40 years and younger 1, 2. BC is characterized with unique tumor microenvironment (TME), associated with promotion of proliferation, metastasis, inhibition of immunological suppression 3. Actually, TME acts as a various ecological niche including heterogeneous clones of tumor cells and other cells, such as endothelial cells, fibroblasts and diverse immune cells and immunosuppressive cells 4. With the development of TME research field, strategies in interventional clinical trials or approved by the FDA to target TME have been regarded as a promising tool for cancer treatment, *e.g.*, Ipilimumab, durvalumab, BLZ945, CDX-301 and so forth 5. However, TME has also been found to mediate diverse drug resistances due to continuous crosstalks by tumor cells and stroma 6. Therefore, further unravelling the potential molecular mechanisms of TME in BC would help us to develop novel treatments.

Solute Carrier Family 3 Member 2 (SLC3A2), also known as CD98, CD98hc or 4F2hc, is an 80 kDa type II transmembrane protein with most of its structure exposed to the extracellular space 7. As one member of system xc^-^, SLC3A2 has been found involved in ferroptosis, metabolism and proliferation of several tumors, including BC, colorectal cancer, melanoma, gastroenteropancreatic-neuroendocrine neoplasm, leukaemia and prostate cancer 8-13. Specifically, Wang et al. unveiled that tumour-associated neutrophils secreted AGR2 increased xCT activity in a SLC3A2-dependent manner, thus promoting metastasis of colorectal cancer among TME 9. Based on the above-mentioned findings, a comprehensive analysis of pathogenic and immunological roles of SLC3A2 in BC is the rationalization for the present study.

It is well known that everolimus works through the combination of everolimus-FK506-binding protein 1A (FKBP1A) complex and mTOR preventing from downstream signaling needed for cell growth 14. In fact, it was initially characterized that FKBP1A inhibits T lymphocytes activation by binding the immunosuppressants FK506^15^. As the smallest and most extensively studied protein among the 18 identified human FK506-binding proteins (FKBPs), FKBP1A exhibited many other functions including binding to different cellular receptors or targets 16-19. Nevertheless, the current role of FKBP1A in TME is still in its infancy for almost all kinds of tumors including BC.

In 2008, Veldhoen and Dardalhon reported that TGF-beta and IL-4 induced the generation of IL-9(+) IL-10(+) Foxp3(-) effector T cells, namely Th9 cells 20. Researchers found that Th9 cells not only contributed to rheumatoid arthritis and systemic lupus erythematosus, but were involved in diverse solid tumors 21. Some studies have preliminarily found that IRF4, GATA-3 and STAT6 were correlated with Th9 cells differentiation 22, 23. Yi et al. demonstrated that tumor-specific Th9 cells could be used for adoptive cancer therapy 24, therefore, it is necessary to further elucidate the underlying molecular mechanism of Th9 cells function and explore a new approach for promoting the efficacy of Th9-based cancer immunotherapy.

In this study, for the first time, we described that both SLC3A2 and FKBP1A were aberrantly expressed among of various BC cohorts. Further results suggested that SLC3A2 and FKBP1A were associated with chemotherapeutic outcome in BC patients. Our results also indicated that SLC3A2 was associated with tumor infiltration of cytotoxic T cell among BC TME. The alterations in SLC3A2 and FKBP1A genes both contributed a significant impact on BC tumor mutational burden. Moreover, SLC3A2 was illustrated expressed in diverse BC cellular populations at single cell level, and negatively linked to angiogenesis, inflammation and quiescence, but positively correlated with other functional phenotypes. In terms of mechanism, FKBP1A was found to bind with SLC3A2, and negatively regulated SLC3A2 expression during the processes of everolimus inducing ferroptosis of BC cells and promoting anti-proliferation of Th9 cells. Identification of FKBP1A/SLC3A2 axis would provide in-depth insights for TME-targeted strategies for BC treatments.

## Results

### SLC3A2 localization, expression and association with breast cancer

**Figure 1:**
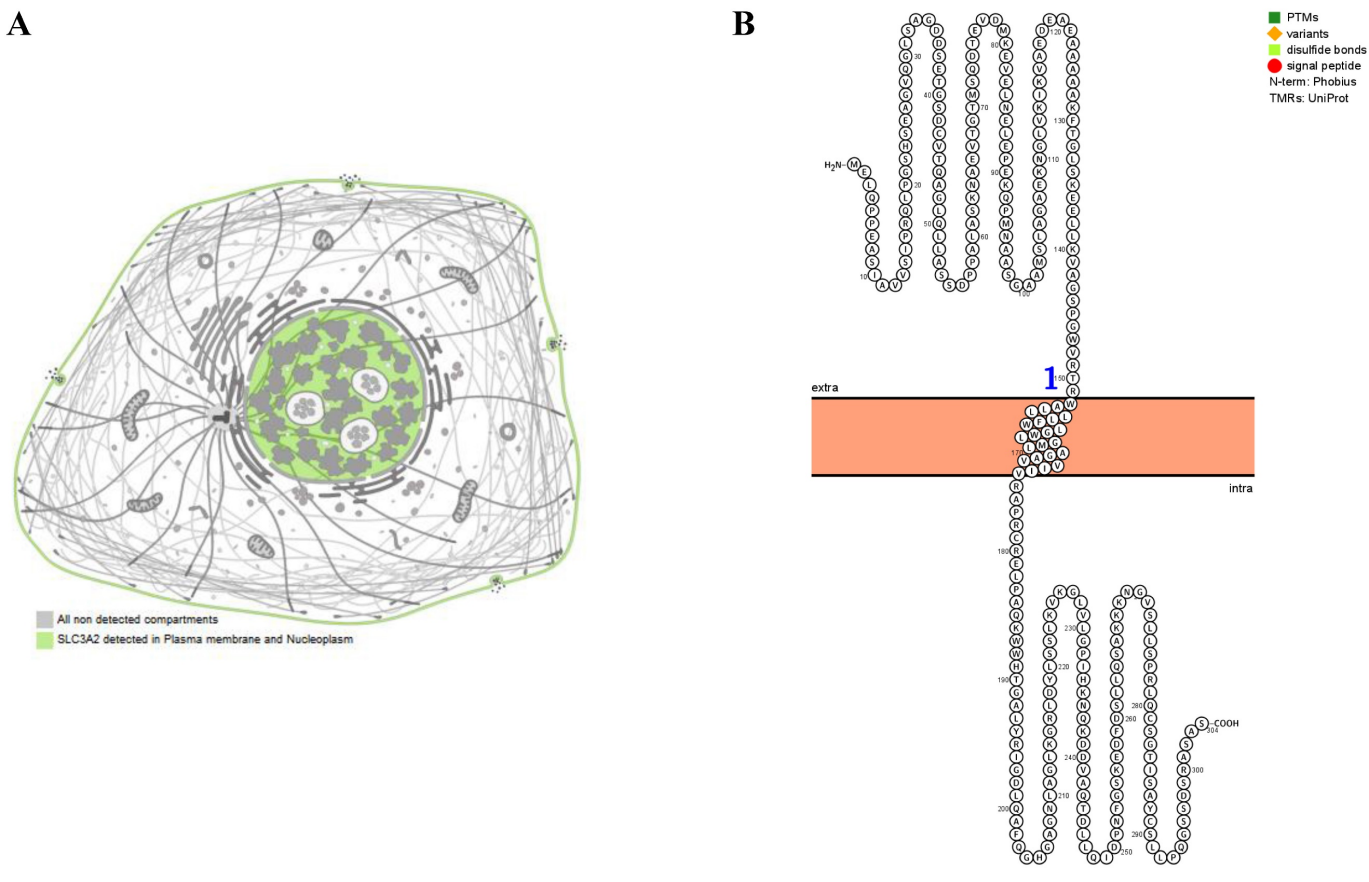

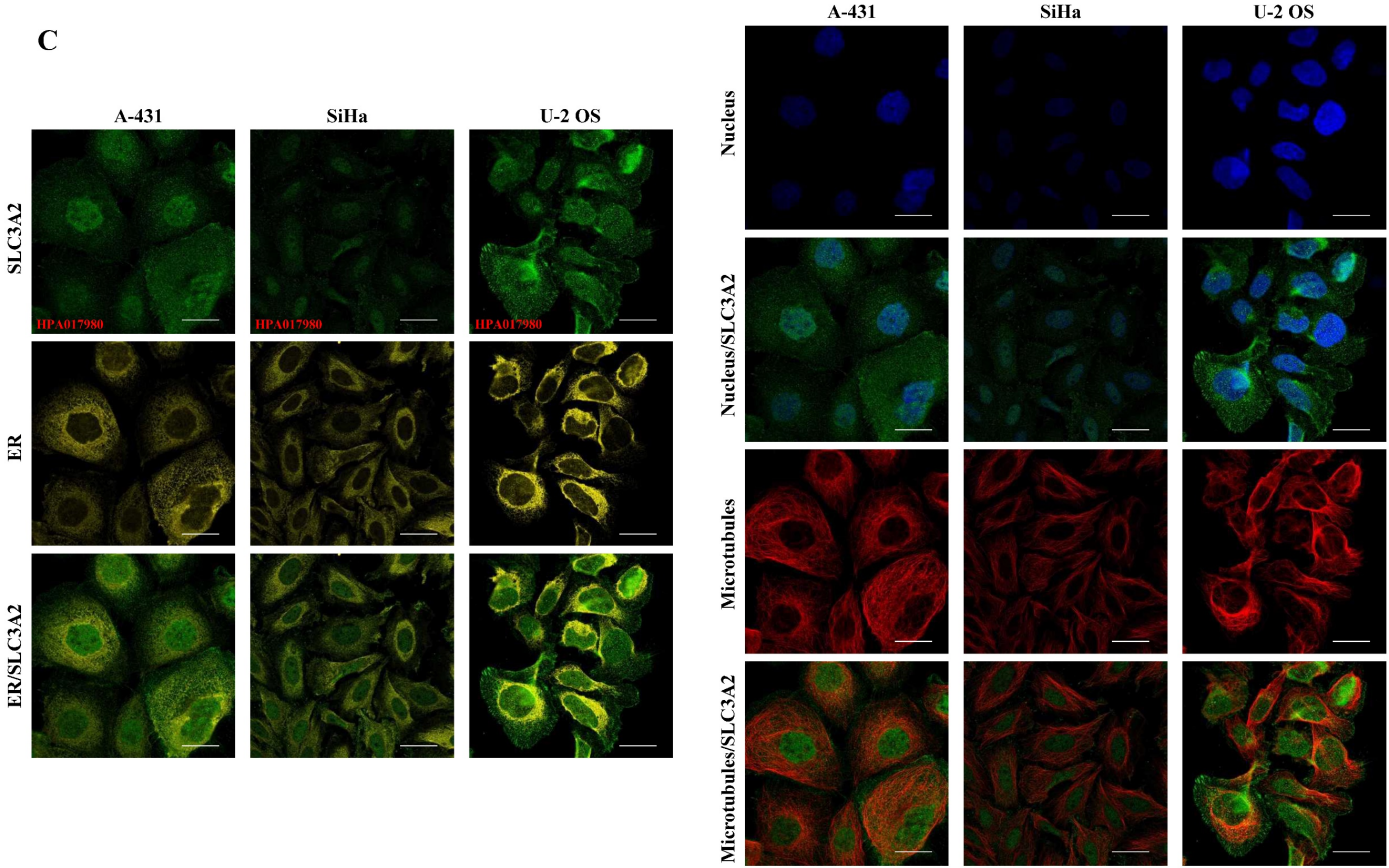

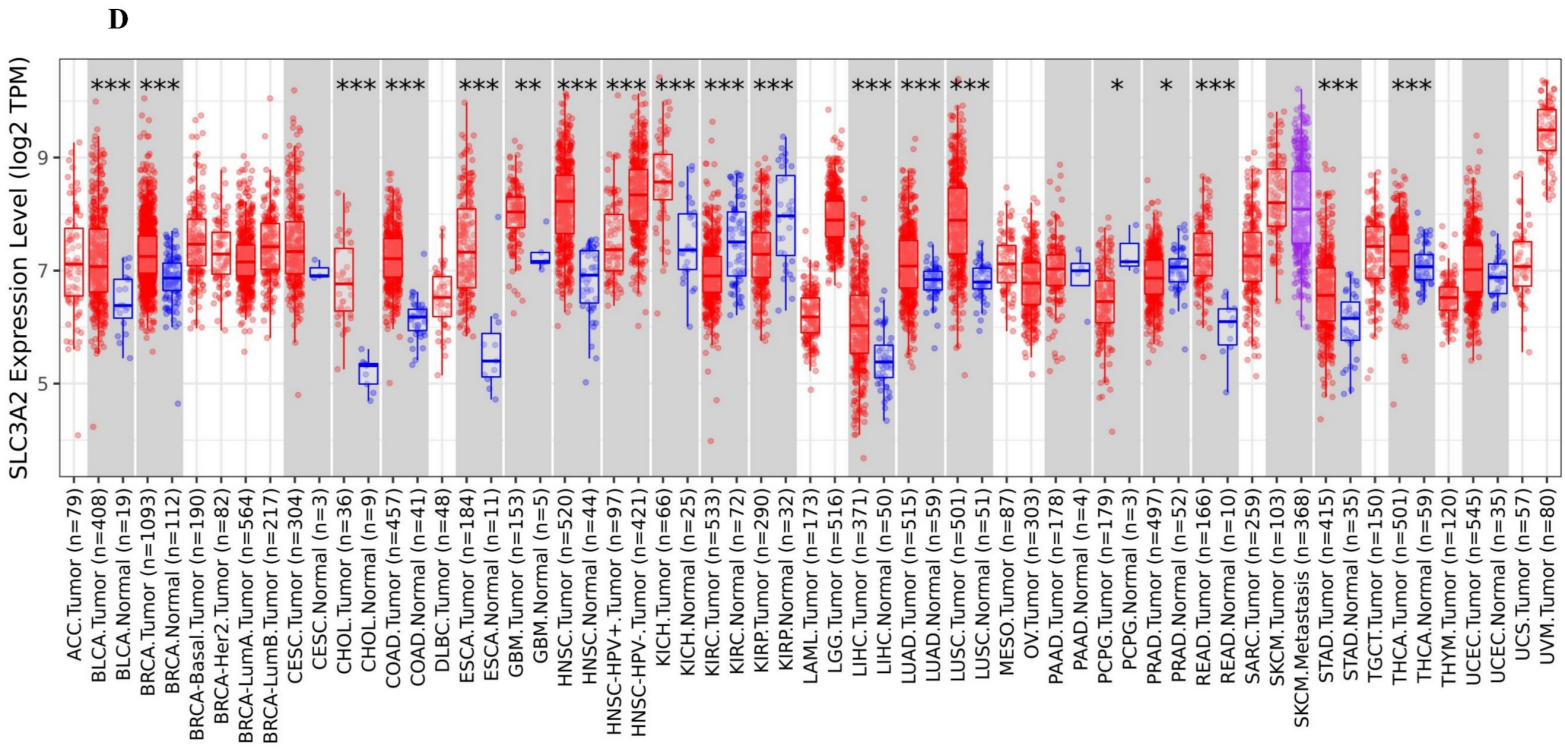

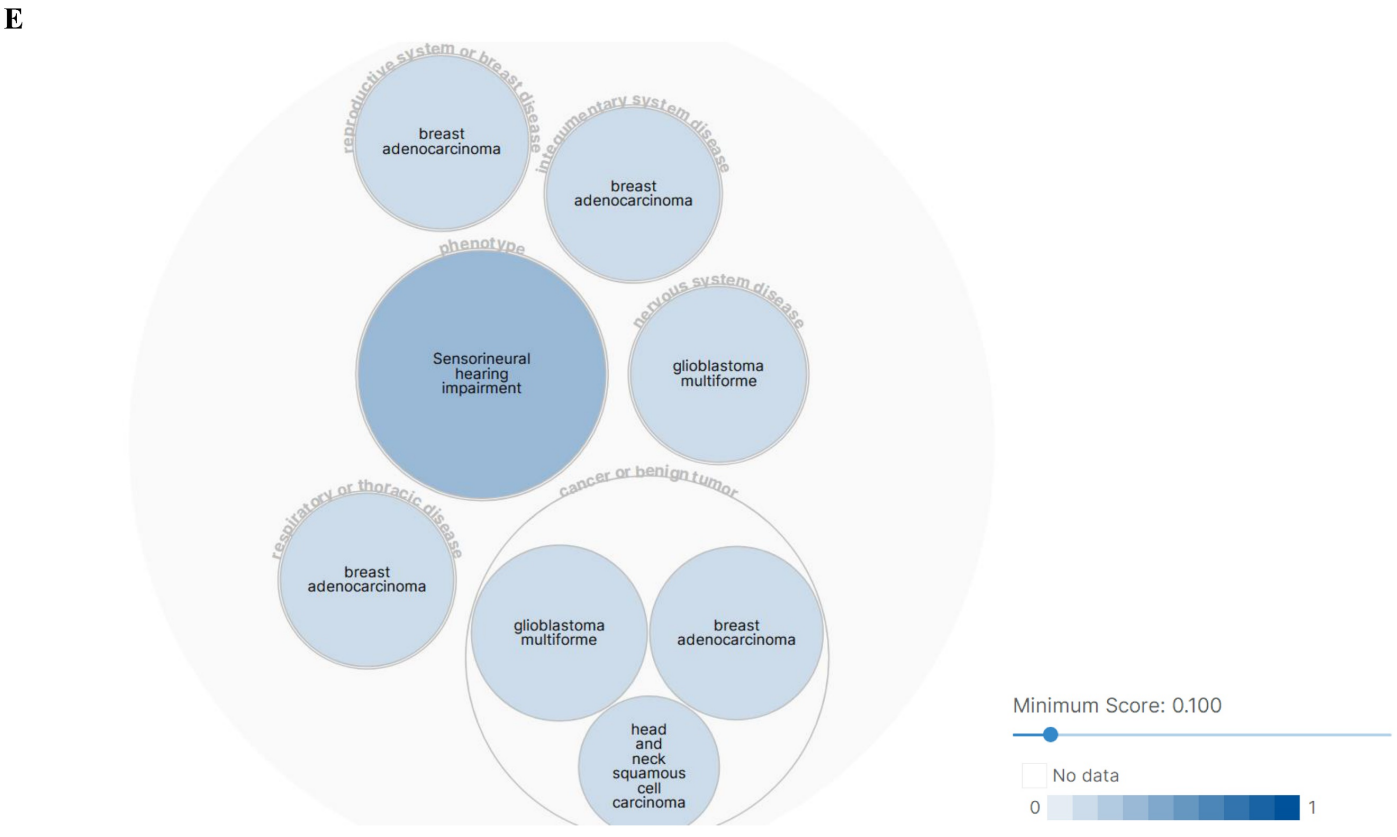

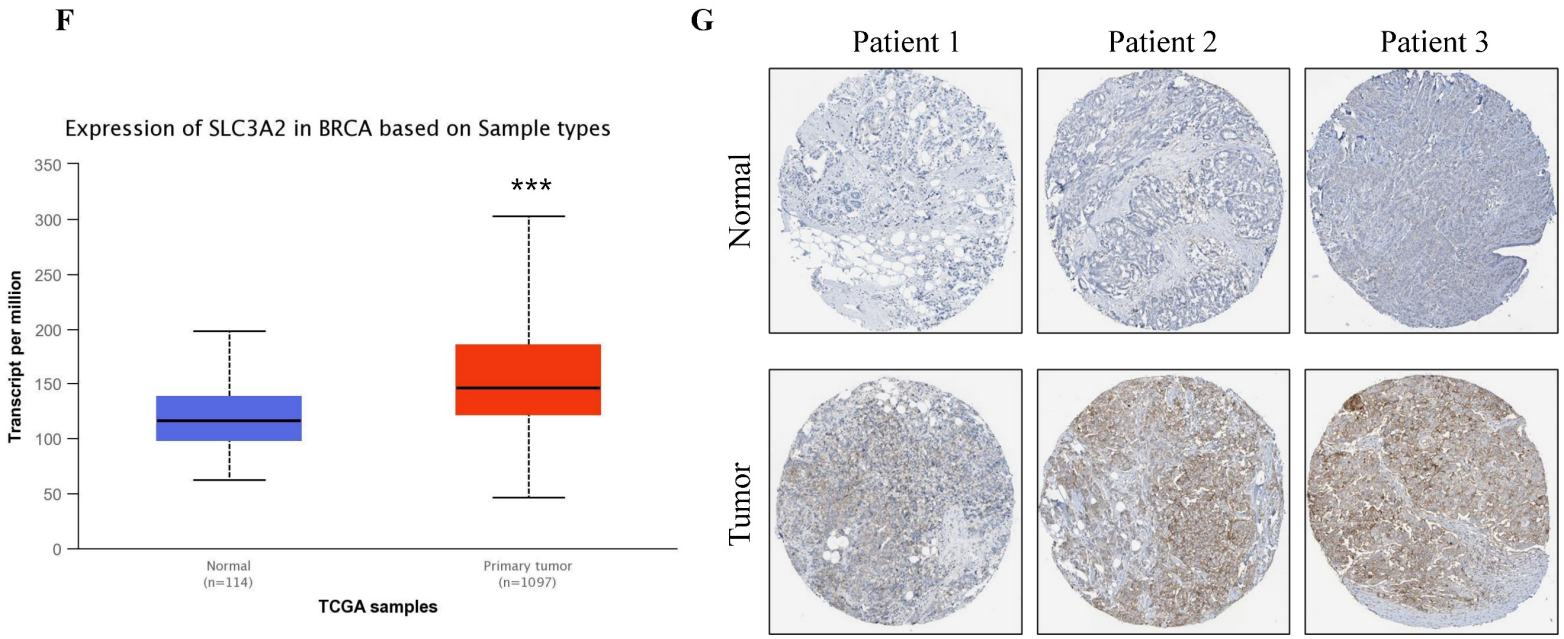
** SLC3A2 localization, expression and association with breast cancer. A** SLC3A2 protein localization showed plasma membrane and nucleoplasm. **B** SLC3A2 protein topology showed crossing cell membrane once. **C** Immunofluorescence staining of the subcellular distribution of SLC3A2 in A-431, SiHa, and U-2 OS cells as adopted from the HPA database. Scale bar = 5 um. **D** The mRNA levels of SLC3A2 in different types of human cancers from TIMER2 database. **E** Disease network interaction analysis of SLC3A2 from OPENTARGET database. **F** The mRNA level of SLC3A2 in breast cancer from UALCAN database. **G** Immunohistochemistry analysis of SLC3A2 in breast cancer from HPA database. *p<0.05, **p<0.01, ***p<0.001.

The SLC3A2 protein was only detected in plasma membrane and nucleoplasm (Fig. 1A), and topology revealed it crossed cell membrane once (Fig. 1B). To show the intracellular localization of SLC3A2, using HPA database, we found that SLC3A2 mainly colocalized with the endoplasmic reticulum (ER) and nucleus marker in A-431, SiHa, and U-2 OS cells (Fig. 1C). Furthermore, we found upregulated SLC3A2 mRNA expression in 14 types of human cancer compared with normal human tissues (Fig. 1D). Since disease network interaction analysis of SLC3A2 from OPENTARGET database showed SLC3A2 strongly associated with breast cancer (BC) (Fig. 1E), we aimed to explore the biological function of SLC3A2 in BC. Both UALCAN and HPA database revealed that SLC3A2 was significantly upregulated in BC patients at mRNA and protein level (Fig. 1F, G), thus playing a potential oncogenic role in BC progression.

### Relation of SLC3A2 and therapeutic responses in breast cancer

Since BC therapeutic backbones include endocrine therapy, anti-HER2 therapy, and chemotherapy 25, we continued to evaluate the effect of SLC3A2 expression on above-mentioned therapeutic responses in clinical cancer cohorts. Taking pathological complete response as the endpoint, we found that higher SLC3A2 expression was not resistant to endocrine therapy, anti-HER2 therapy, or chemotherapy (Fig. S1A-C), while BC patients with higher SLC3A2 expression benefitted less from chemotherapy when relapse-free survival at 5 years was considered (Fig. S1D-F). These results implied that SLC3A2 could act as an adverse therapeutic factor in BC.

### Relation of SLC3A2 and clinicopathological characteristics in breast cancer

In UALCAN database, we found SLC3A2 expression in male BC patient was the top (Fig. S2A). With the increase of cancer stage and nodal metastasis status, SLC3A2 expression continued to rise (Fig. S2B, C). For the molecular subtype, upregulated FKBP1A was significantly related to luminal, HER2 positive and triple negative subtype patients than the normal group (Fig. S2D). All diverse races, ages, menopause status and TP53 mutation status of BC patients had higher level expression of SLC3A2 than the normal group (Fig. S2E-H).

### SLC3A2 is associated with tumor infiltration of cytotoxic T cell but no other immune cells

**Figure 2:**
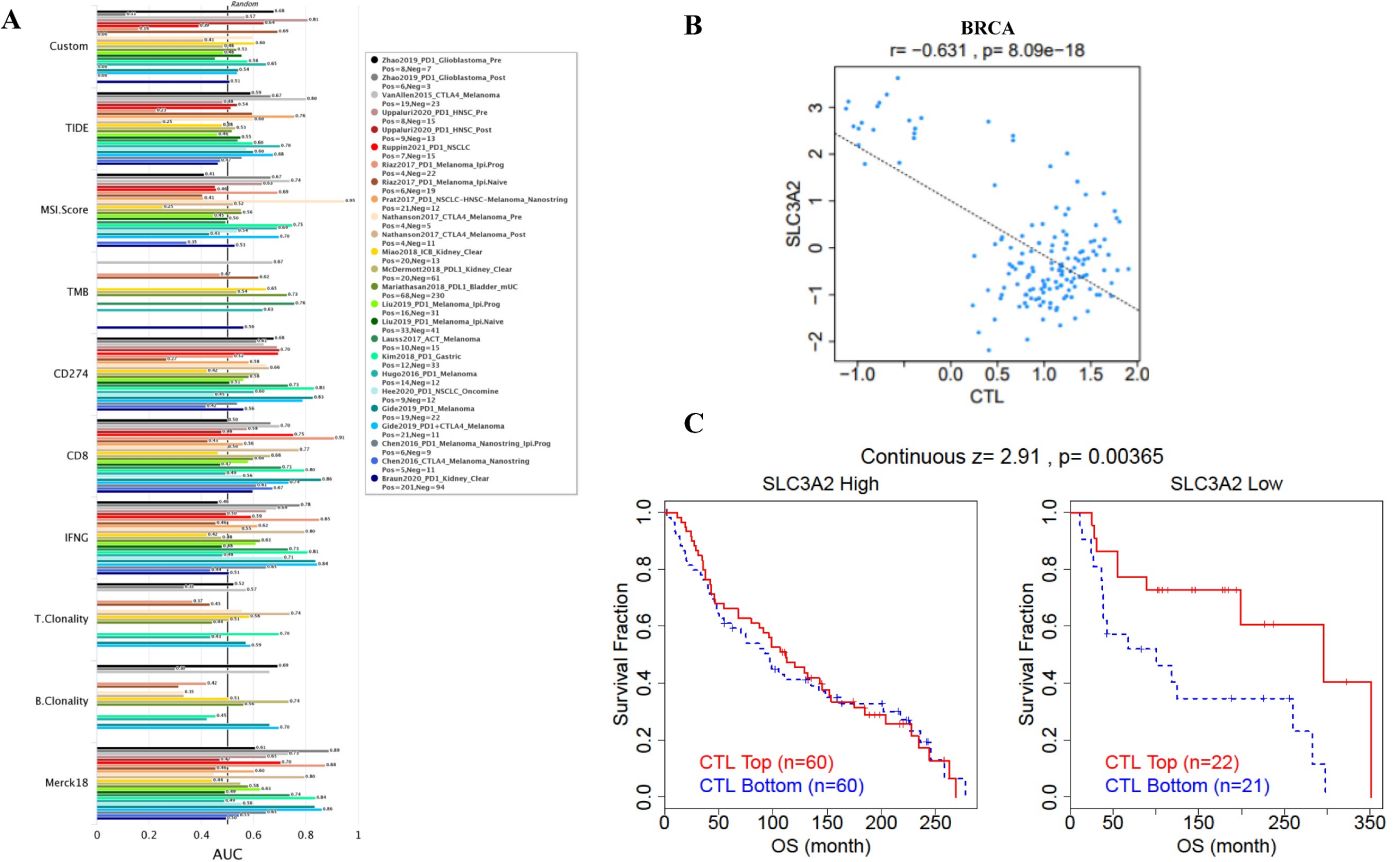

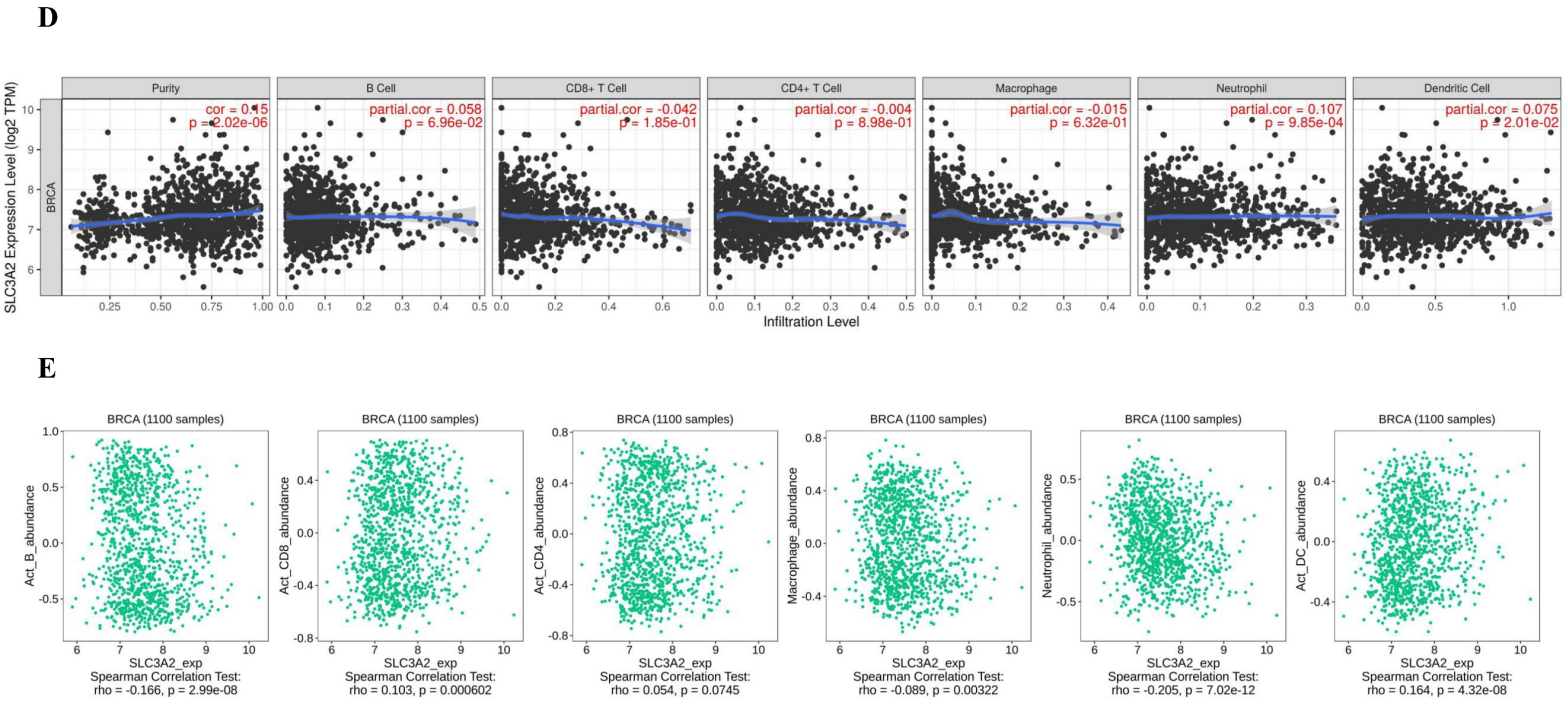

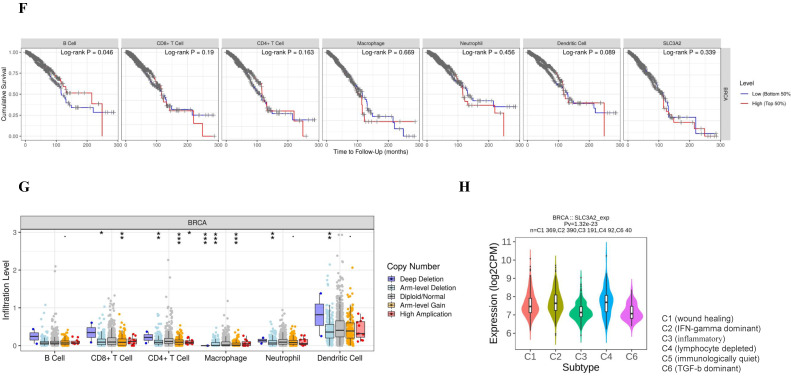

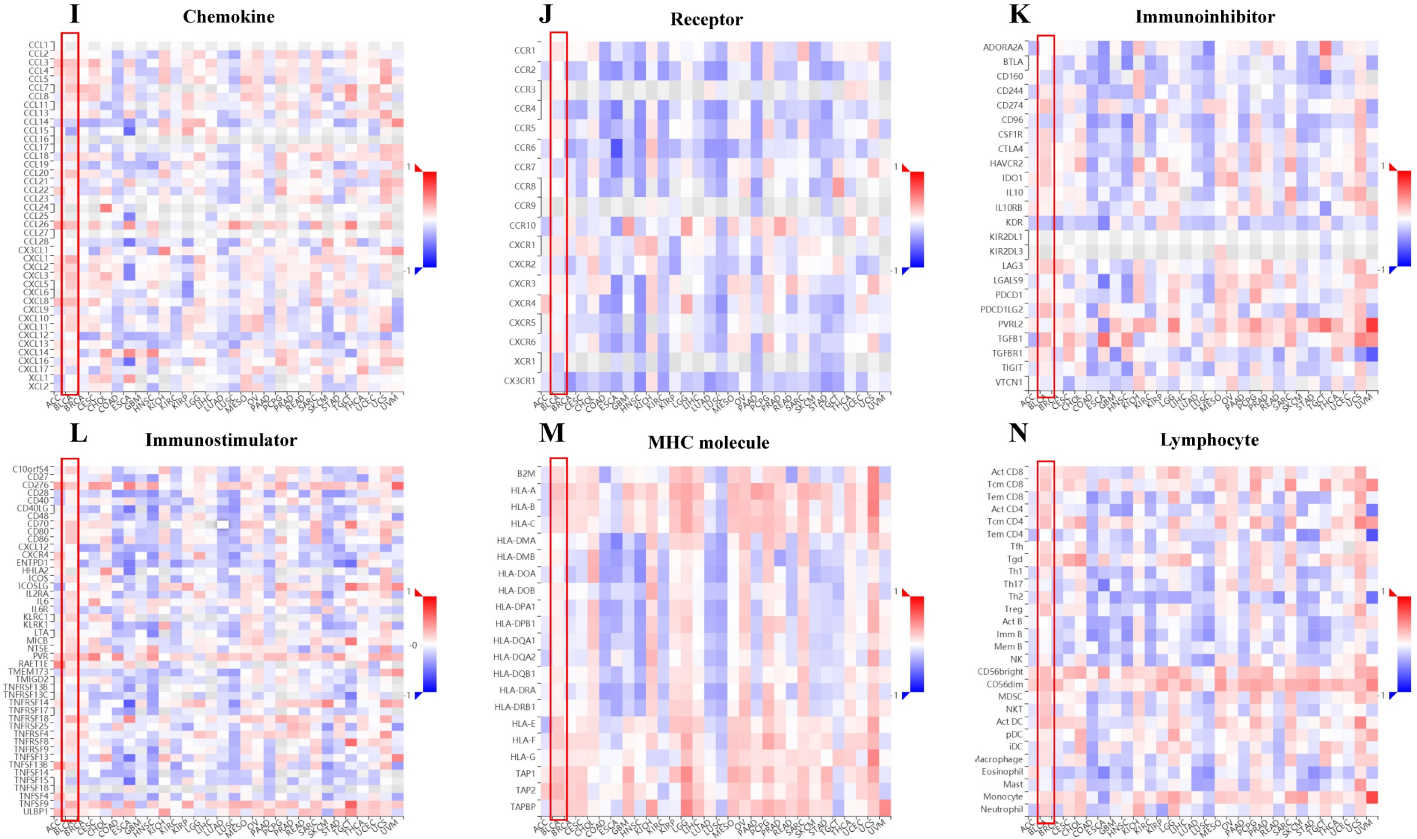
** Relation of SLC3A2 and immunolgy in breast cancer. A** Bar plot showing the biomarker relevance of SLC3A2 compared to standardized cancer immune evasion biomarkers in immune checkpoint blockade sub-cohorts. The area under the receiver operating characteristic curve was applied to evaluate the predictive performances of the test biomarkers on the immune checkpoint blockade response status. **B** Pearson's pairwise correlation plot of SLC3A2 and CTL infiltration in breast cancer patients, r= -0.631, p<0.001. **C** Kaplan-Meier survival curves of CTL infiltration in SLC3A2 high or low BC patients. **D** Spearman correlations between expression of SLC3A2 and infiltration level of diverse immune cells in breast cancer using TIMER database. **E** Spearman correlations between expression of SLC3A2 and infiltration level of diverse immune cells in breast cancer using TISIDB database. **F** Survival analysis of SLC3A2 expression and diverse immune cells infiltration level in breast cancer. **G** Comparison of diverse immune cell infiltration levels among breast cancer with different somatic copy number alterations for SLC3A2 using TIMER database. **H** Distribution of SLC3A2 expression across immune subtypes in breast cancer using TISIDB database. **I-N** Heatmap of correlation analyses of the SLC3A2 expression with chemokines, receptors, immunoinhibitors, immunostimulators, MHC molecules and lymphocytes from TISIDB database in breast cancer. *p<0.05, **p<0.01, ***p<0.001.

Immune cells among TME are associated with the hallmarks of BC 3, we continued to evaluate the biomarker relevance of SLC3A2 compared to standardized cancer biomarkers in immune checkpoint blockade sub-cohorts using TIDE database. Results showed that SLC3A2 alone had an area under the receiver operating characteristic curve of > 0.5 in 12 of the 25 immune checkpoint blockade sub-cohorts (Fig. 2A). Among various immune cells, we found SLC3A2 was only significantly negatively correlated with cytotoxic T cell (CTL) in BC TME (Fig. 2B). Meanwhile, we demonstrated that only in BC patients with low SLC3A2 expression, the higher expression of CTL was positively correlated with patients' OS (Fig. 2C), suggesting that SLC3A2 might exert biological effects by affecting CTL infiltration in BC patients. In both TIMER and TISIDB database, although we found SLC3A2 was closely associated with numerous immune cell infiltrations (Fig. 2D, E), it almost did not affect the role of these immune cells in patient prognosis (Fig. 2F). Fig. 2G showed remarkable relations of the changes in SLC3A2 copy number variation and infiltration level in BC, specifically, arm-level deletion of SLC3A2 was significantly connected with five immune infiltration levels. In addition to SLC3A2's role in immune typing of BC (Fig. 2H), we found that SLC3A2 remarkably correlated with most chemokines, receptors, immunoinhibitors, immunostimulators, MHC molecules and lymphocytes (Fig. 2I-N). These findings implied the potential role of SLC3A2 to predict the immune therapy response in BC.

### Relation of SLC3A2 and genetic alterations in breast cancer

**Figure 3:**
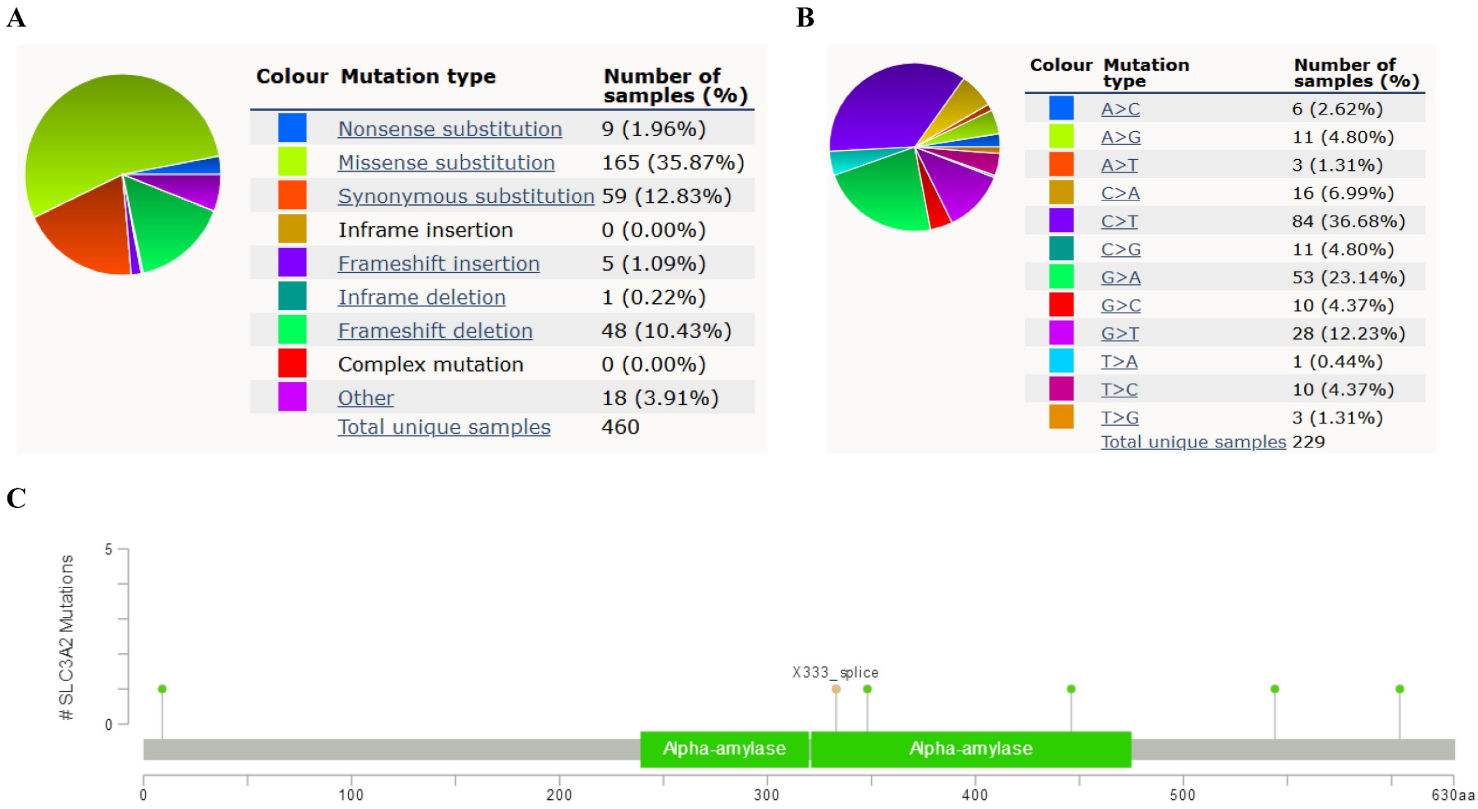

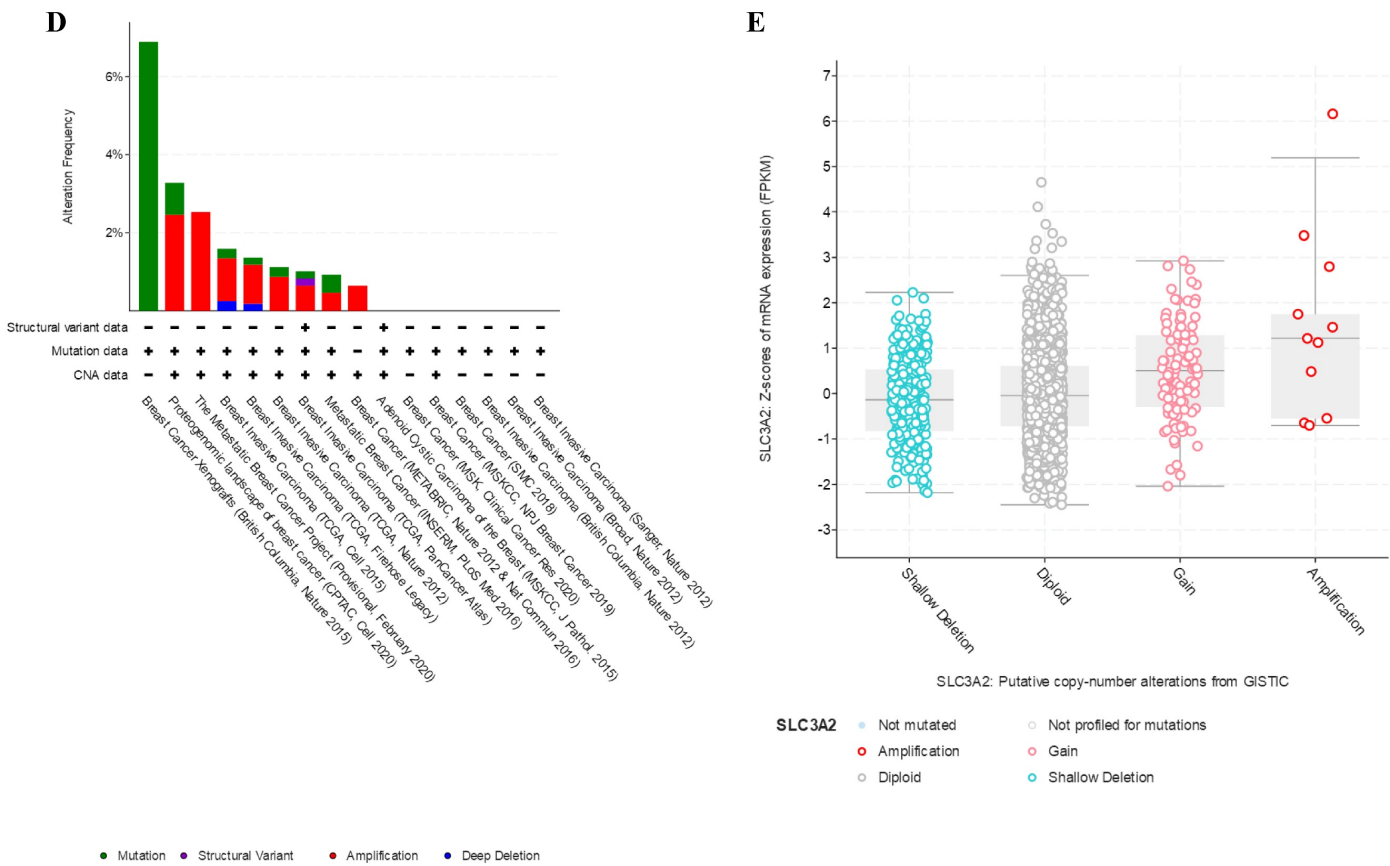

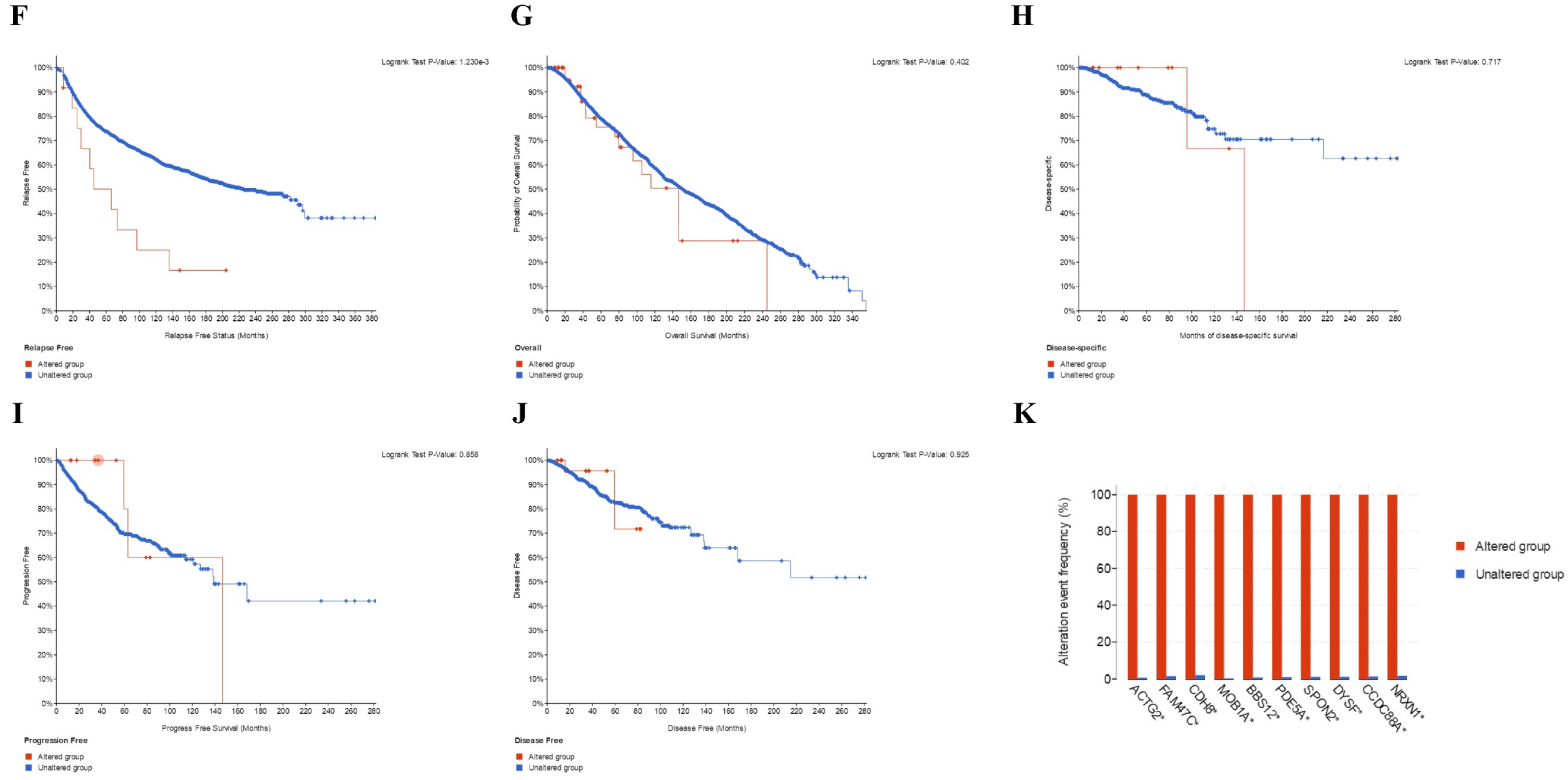

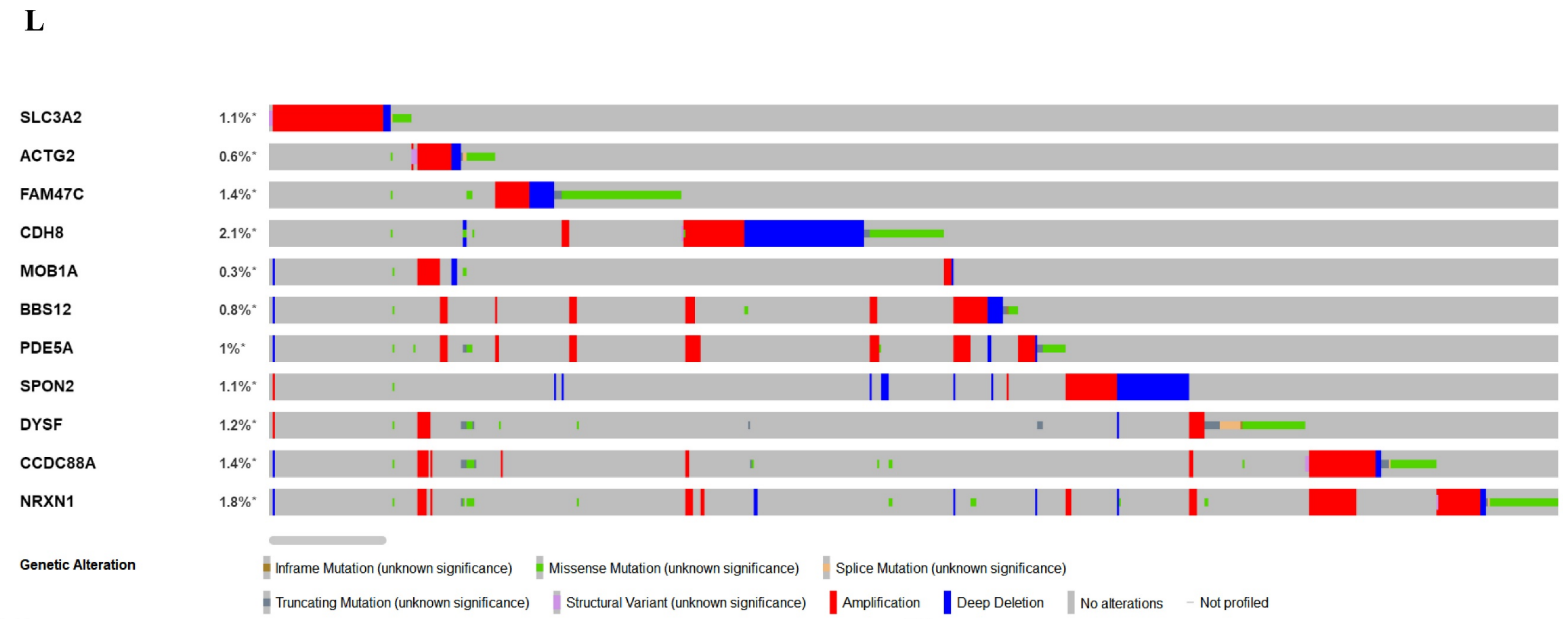

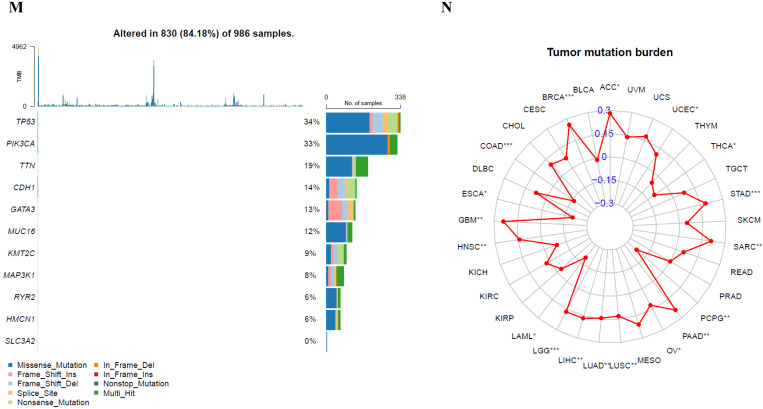
** Relation of SLC3A2 and genetic alterations in breast cancer. A-B** Pie-chart showed the percentage of the mutation type of SLC3A2 in breast cancer according to COSMIC database. **C** SLC3A2 mutation sites at protein level according to cBioPortal database. **D** Oncoprint in cBioPortal represented the proportion and distribution of samples with alterations in SLC3A2 gene.** E** Putative copy-number SLC3A2 gene alternations according to cBioPortal database. **F-J** Kaplan-Meier plots comparing relapse free status, overall survival, months of disease-specific, progress free survival, and disease free in cases with/without SLC3A2 gene alterations. **K** Bar plot showing the frequencies of ACTG2, FAM47C, CDH8, MOB1A, BBS12, PDE5A, SPON2, DYSF, CCDC88A, and NRXN1 alteration co-occurrence with SLC3A2 alterations. **L** Waterfall plot showing the co-occurrence pattern of SLC3A2 alterations with genetic alterations of ACTG2, FAM47C, CDH8, MOB1A, BBS12, PDE5A, SPON2, DYSF, CCDC88A, and NRXN1. **M** Waterfall plot showing SLC3A2 mutations compared with common mutated genes in TCGA database. **N** Radar map of connection of SLC3A2 and tumor mutation burden in pan-cancer. *p<0.05, **p<0.01, ***p<0.001.

In COSMIC database, the pie chart included various mutations information including nonsense substitution, missense substitution, synonymous substitution, etc. The proportion of missense substitution was the highest (35.87%) (Fig. 3A). BC mainly had 36.68% C > T, 23.14% G > A and 12.23% G > T mutation in SLC3A2 coding strand (Fig. 3B). At the protein structure level, SLC3A2 mutation sites were concentrated in the 300-630AA region (Fig. 3C). Alteration frequency of SLC3A2 mutation in BC was analyzed by using cBioPortal. From 1% to 7% mutation in the patients with BC was observed (Fig. 3D). At the mRNA level, SLC3A2 mutation forms were concentrated in the form of gain and amplification (Fig. 3E). After analyzed by Kaplan-Meier plot and log-rank test, the alterations in SLC3A2 had a significant correlation to relapse free survival but no other forms of survival in BC patients (Fig. 3F-J). We then found that SLC3A2 alteration was perfectly correlated with several other genes expression, including ACTG2, FAM47C, CDH8, MOB1A, BBS12, PDE5A, SPON2, DYSF, CCDC88A, and NRXN1 (Fig. 3K, L). Although SLC3A2 had little change compared with common mutated genes in TCGA database (Fig. 3M), it contributed a significant impact on BC tumor mutational burden (Fig. 3N).

### Relation of SLC3A2 and methylation in breast cancer

**Figure 4:**
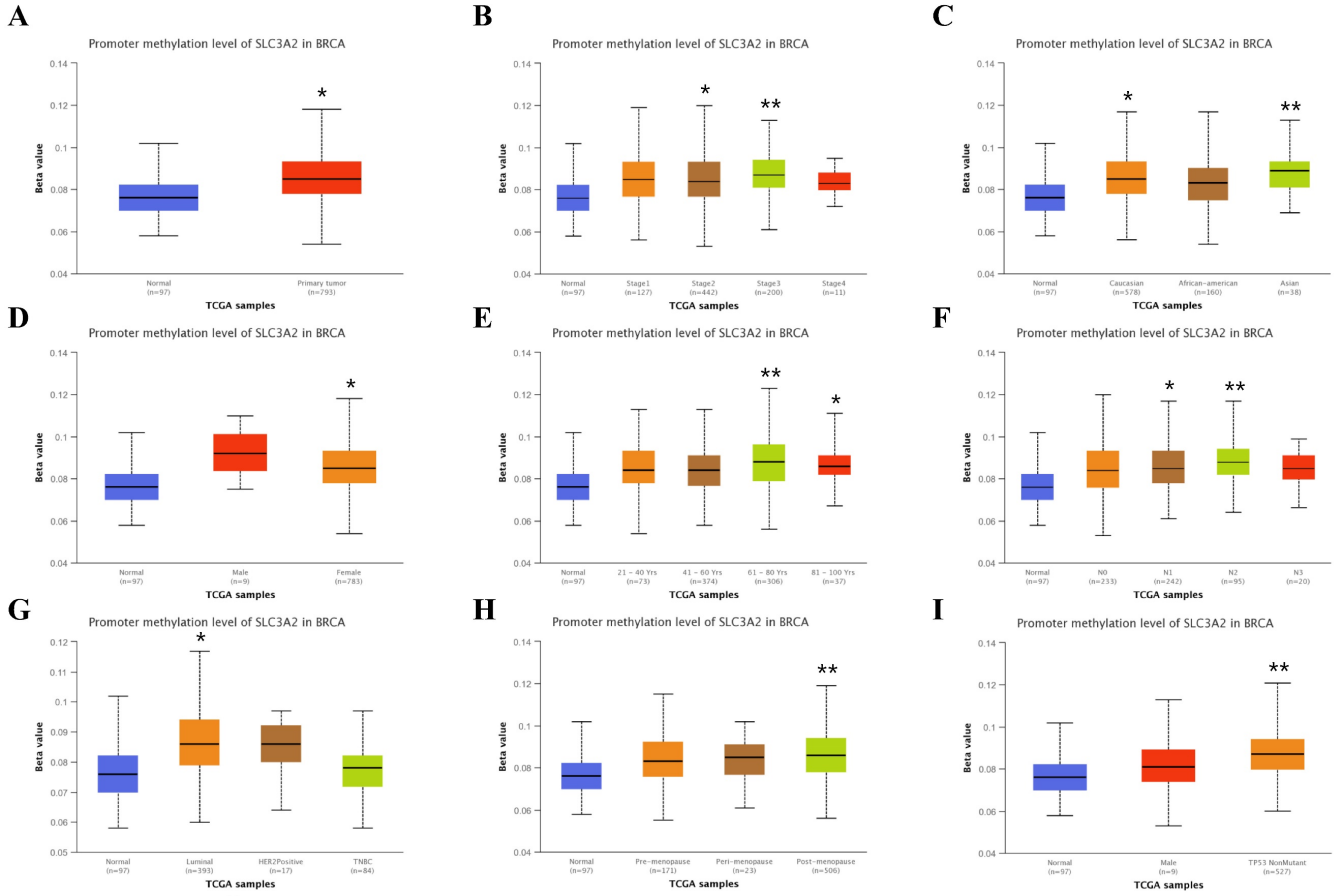

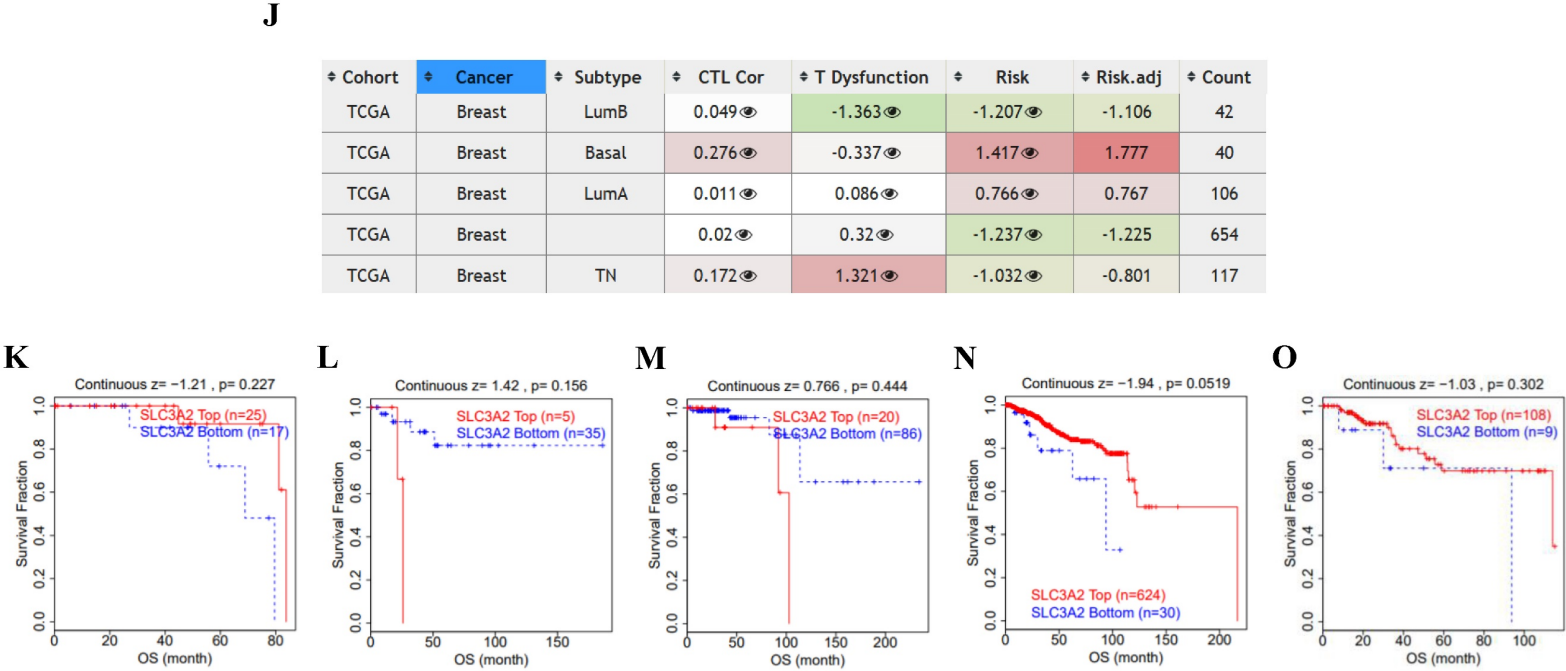
** Relation of SLC3A2 and methylation in breast cancer. A-I** Correlations between SLC3A2 gene promoter methylation level and tumor samples, cancer stages, races, genders, ages, nodal metastasis status, molecular subtypes, menopause status and TP53 mutation status. **J** Heatmap showing the roles of SLC3A2 methylation in cytotoxic T-cell levels, dysfunctional T-cell phenotypes, and risk factors of TCGA cancer cohorts. **K-O** Kaplan-Meier curves of overall survival differences between five cytotoxic T-cell related TCGA cancer cohorts with high methylation levels and those with low methylation levels of SLC3A2. *p<0.05, **p<0.01.

Recent researchers have found the indispensable role of epigenetic alterations including methylation in the mechanism of BC pathogenesis 26, using the UALCAN and TIDE database, we then explored the DNA methylation of SLC3A2. Results showed SLC3A2 gene promoter methylation level was significantly upregulated in BC compared with normal tissues (Fig. 4A). With the increase of cancer stage and nodal metastasis status, SLC3A2 gene promoter methylation level continued to rise (Fig. 4B, F). For the molecular subtype, upregulated SLC3A2 gene promoter methylation level was only significantly related to luminal subtype patients than the normal group (Fig. 4G). Additionally, diverse races, genders, ages, menopause status and TP53 mutation status of BC patients had higher SLC3A2 gene promoter methylation level than the normal group, respectively (Fig. 4C-E, H, I). As for the hypermethylation of SLC3A2 associated with CTL, results showed no obvious correlation (Fig. 4J), and SLC3A2 gene promoter methylation level referred to no significantly longer survival durations among various molecular subtypes of BC patients (Fig. 4K-O).

### Relation of SLC3A2 and single cell sequencing in breast cancer

To acquire a deeper comprehension of the possible function of SLC3A2 in BC at single cell level, further analysis of the correlation of SLC3A2 functional states was undertaken among four single cell datasets (EXP0052, EXP0053, EXP0054, EXP0055) utilizing the CancerSEA database (Fig. S3A). In EXP0052, SLC3A2 expression was significantly various in multiple functional phenotypes, such as apoptosis, hypoxia and metastasis (Fig. S3A-C). In EXP0053, SLC3A2 expression was significantly various in multiple functional phenotypes, such as apoptosis, DNA damage and invasion (Fig. S3A, D, E). In EXP0054, SLC3A2 expression was significantly various in multiple functional phenotypes, such as angiogenesis, apoptosis, cell cycle, hypoxia, DNA damage, EMT, hypoxia, DNA repair, invasion, metastasis and proliferation (Fig. S3A, F, G). In EXP0055, SLC3A2 expression was significantly various in multiple functional phenotypes, such as inflammation, proliferation and quiescence (Fig. S3A, H, I). In conclusion, SLC3A2 was expressed in diverse BC cellular populations, and negatively linked to angiogenesis, inflammation and quiescence, but positively correlated with other functional phenotypes.

### FKBP1A/SLC3A2 axis in everolimus inducing ferroptosis of breast cancer and anti-proliferation of T lymphocyte

**Figure 5:**
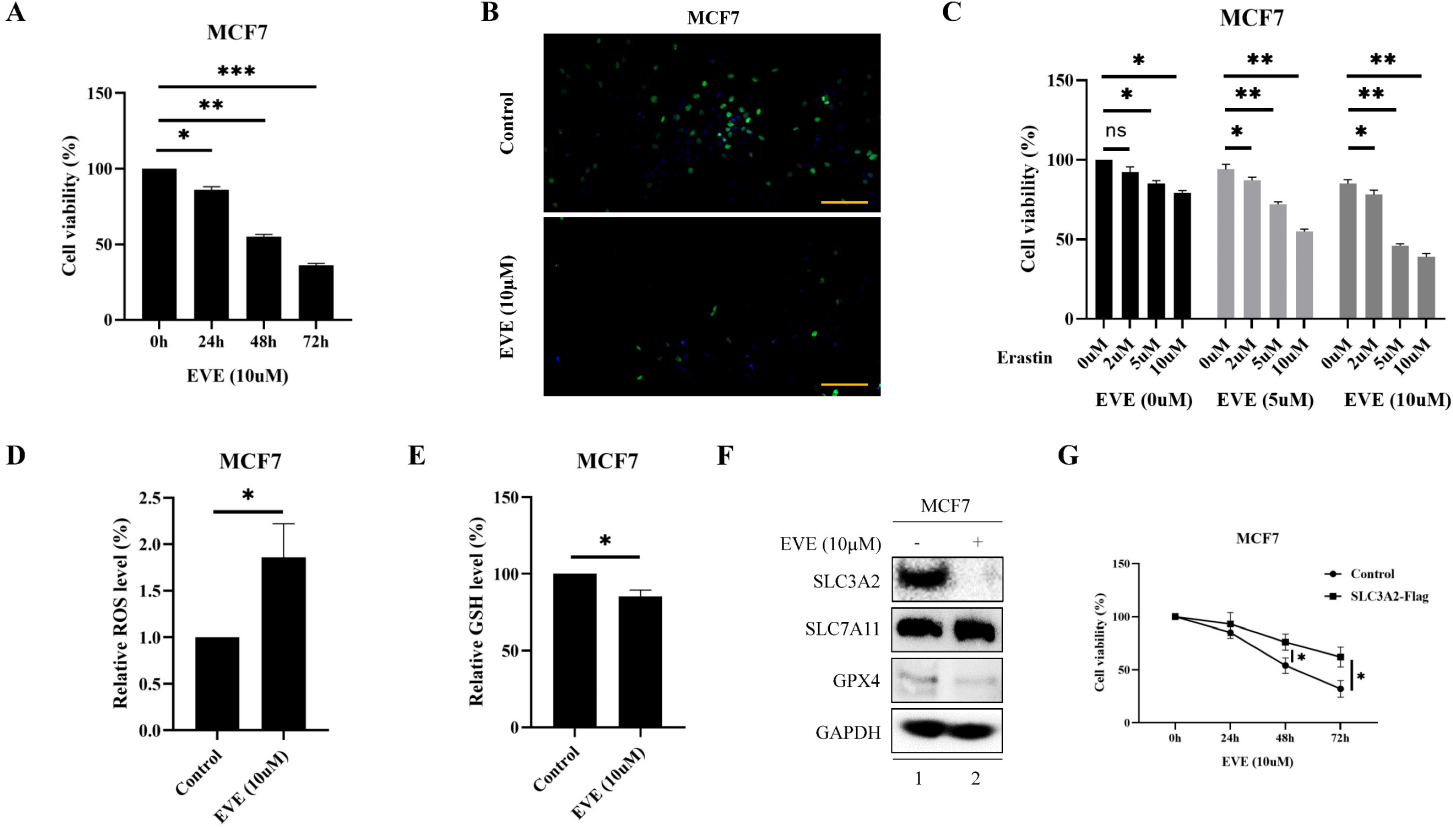

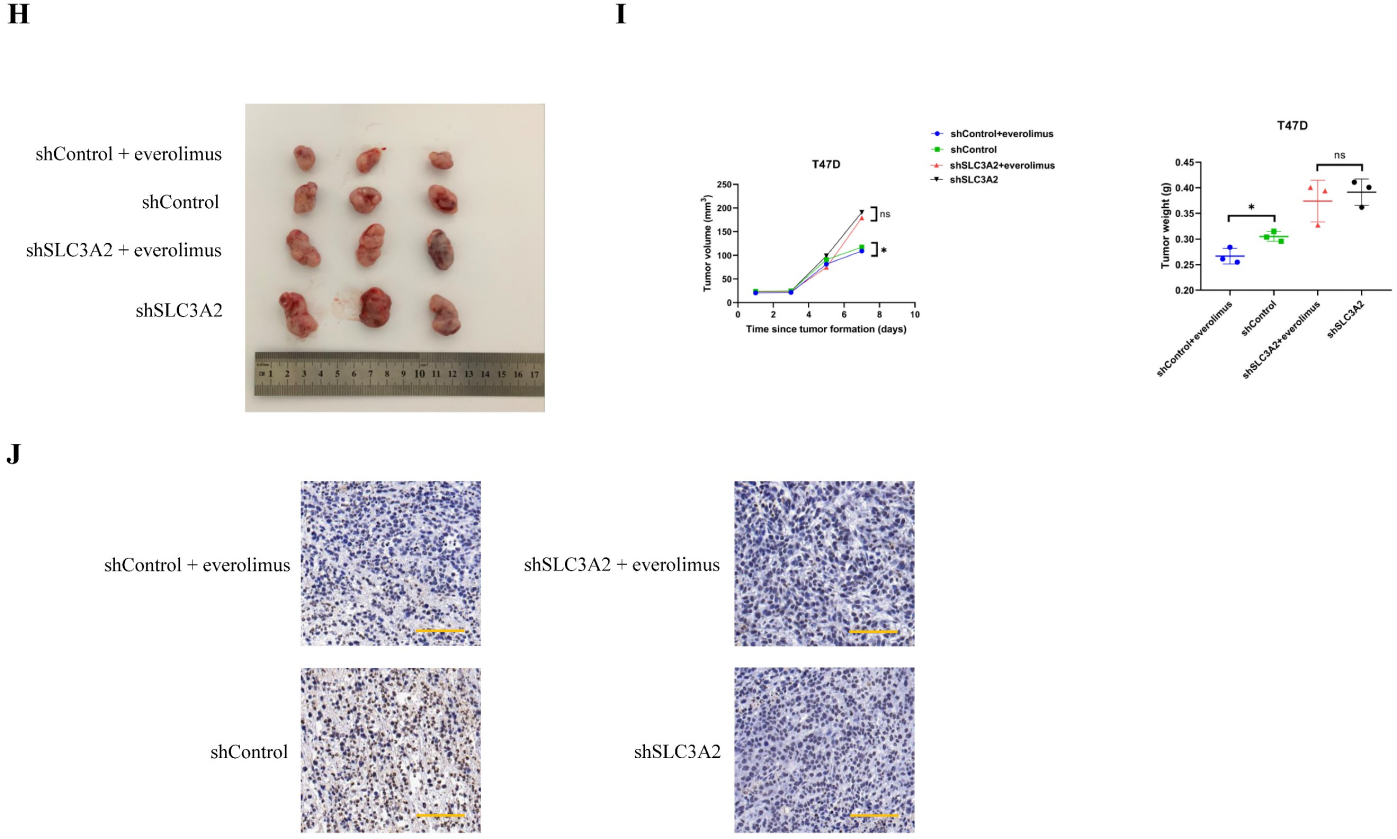

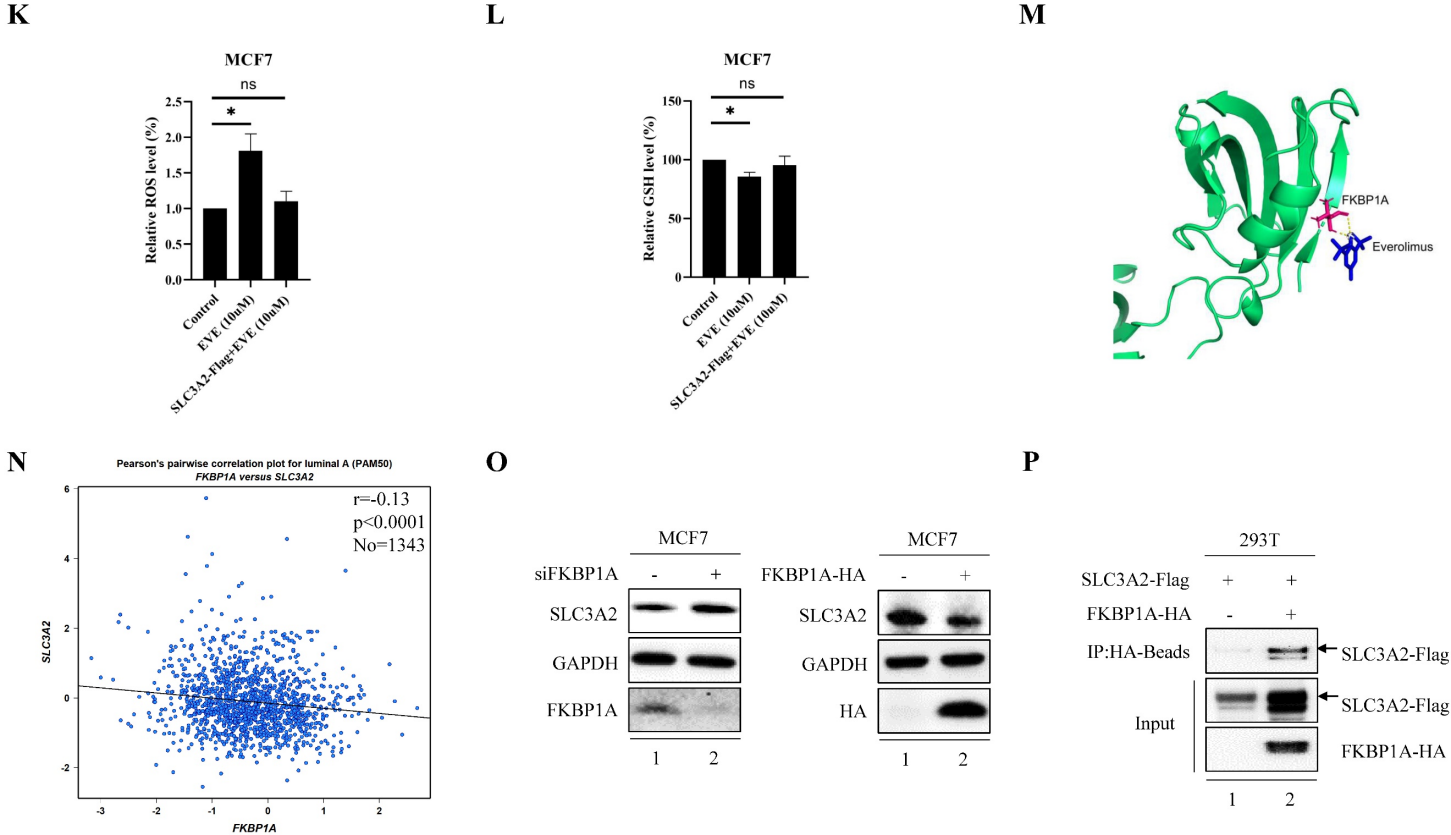
** FKBP1A/SLC3A2 axis in everolimus inducing ferroptosis of breast cancer cells. A** MCF7 cells were treated with everolimus (10 uM) for 0, 24, 48, or 72 h, and cell viability was assayed. **B** Comparison of MCF7 cell proliferative capacity among control and everolimus (10 uM) groups at 72 h via Edu staining. Scale bar = 50 um. **C** MCF7 cells were treated with varying doses of everolimus alone or in combination with different doses of erastin for 48 h; cell viabilities were measured. **D** MCF7 cells were treated with everolimus (10 uM) for 48 h. The relative total ROS levels were assayed via DCFH-DA fluorescence. **E** MCF7 cells were treated with everolimus (10 uM) for 48 h, and the relative levels of GSH were assayed. **F** MCF7 cells were treated with everolimus (10 uM) for 72 h. SLC3A2, SLC7A11 and GPX4 protein expression were measured via Western blotting. **G** MCF7 cells were treated with everolimus (10 uM) for 0, 24, 48, or 72 h with or without the overexpression of SLC3A2. Cell viability was assayed. **H, I** The macroscopic appearance, volume and weight of subcutaneous tumor in mice (n = 3/group) transplanted with T47D cells treated with shControl + everolimus, shControl, shSLC3A2 + everolimus and shSLC3A2. **J** Representative images of immunohistochemical staining (SLC3A2) of T47D cell-derived xenograft tumors with the indicated treatments. Scale bar = 100 um. **K** MCF7 cells were treated with everolimus (10 uM) for 48 h with or without the overexpression of SLC3A2. The relative total ROS levels were assayed via DCFH-DA fluorescence.** L** MCF7 cells were treated with everolimus (10 uM) for 48 h with or without the overexpression of SLC3A2, and the relative levels of GSH were assayed. **M** 3D cartoon showed everolimu (blue) binds to the structure of a part in FKBP1A (red). **N** Pearson's pairwise correlation plot of FKBP1A and SLC3A2 in luminal A subtype of breast cancer patients, r = -0.13, p<0.0001, No = 1343. **O** After knockdown of FKBP1A with siRNA or overexpression of FKBP1A in MCF7 cells, the relative SLC3A2 protein levels were measured via Western blotting. **P** Co-IP and Western blot assays using antibodies as indicated for binding between exogenous FKBP1A and SLC3A2 in 293T cells. *p<0.05, **p<0.01, ***p<0.001, ns=nonsense.

Accumulating evidence has made everolimus approved for the treatment of metastatic ER positive, HER2 negative BC patients, because everolimus could effectively restrain the proliferation via inhibiting serine/ threonine kinase mTOR 2, 27. Consistently, current study also showed that everolimus could effectively inhibit the proliferation of MCF7 and T47D cells in time-dependent manner (Fig. 5A, B, Fig. S4A, B). Since everolimus has been shown to accelerates erastin-induced ferroptosis in renal cell carcinoma 28, we explored whether ferroptosis was involved in the anti-cancer activity of everolimus in BC. The results showed that everolimus-restrained cell viability in MCF7 and T47D cells could be accelerated by erastin (Fig. 5C, Fig. S4C). To further investigate the association between everolimus and ferroptosis, we focused on the biochemical processes of ferroptosis, *e.g.*, total ROS and GSH level. Our results showed that in MCF7 cells everolimus significantly increased intracellular total ROS level (Fig. 5D) and reduced intracellular GSH level (Fig. 5E).

Unsurprisingly, everolimus effectively inhibited the expression of SLC3A2 and GPX4 in MCF7 cells, but had no effects on SLC7A11 (Fig. 5F). To further clarify the role of SLC3A2 in the anti-tumor effect of everolimus, we demonstrated that overexpression of SLC3A2 significantly reversed the anti-tumor effect of everolimus in both MCF7 and T47D cells (Fig. 5G, Fig. S5A), while downexpression of SLC3A2 significantly enchanced the anti-tumor effect of everolimus in both MCF7 and T47D cells (Fig. S5B, C). We then found that overexpression of SLC3A2 also significantly enhanced the downregulation of ROS and the upregulation of GSH (Fig. 5K, L). Additionally, T47D cells treated with silenced SLC3A2 were used to generate subcutaneous xenograft models in nude mice. We found that the volume and weight of tumor were significantly reduced in the everolimus treated group than in the control group, but those in the everolimus + shSLC3A2 group were similar to the shSLC3A2 group (Fig. 5H, I). Moreover, mice treated with everolimus showed lower SLC3A2 expression using immunohistochemistry (Fig. 5J). All together, these results suggested that everolimus might induce ferroptosis partially via downregulating the SLC3A2 protein level.

It is well-known that everolimus and its analogues bind to the intercellular receptor FKBP1A (Fig. 5M), and then predominantly inhibit mTORC1 signaling 29. Based on the above-mentioned findings, we aimed to clarify the effect of FKBP1A on SLC3A2 expression. Using bc-GenExMiner v4.7 database, we found that FKBP1A was negatively correlated with SLC3A2 in luminal A subtype of BC (Fig. 5N). Furthermore, in MCF cells knockdown of FKBP1A was found to promote the protein level of SLC3A2, while overexpression of FKBP1A inhibited the protein level of SLC3A2, and these two proteins could bind to each other (Fig. 5O, P). In summary, our findings indicated that everolimus induced ferroptosis by regulating the FKBP1A/SLC3A2 axis.

**Figure 6:**
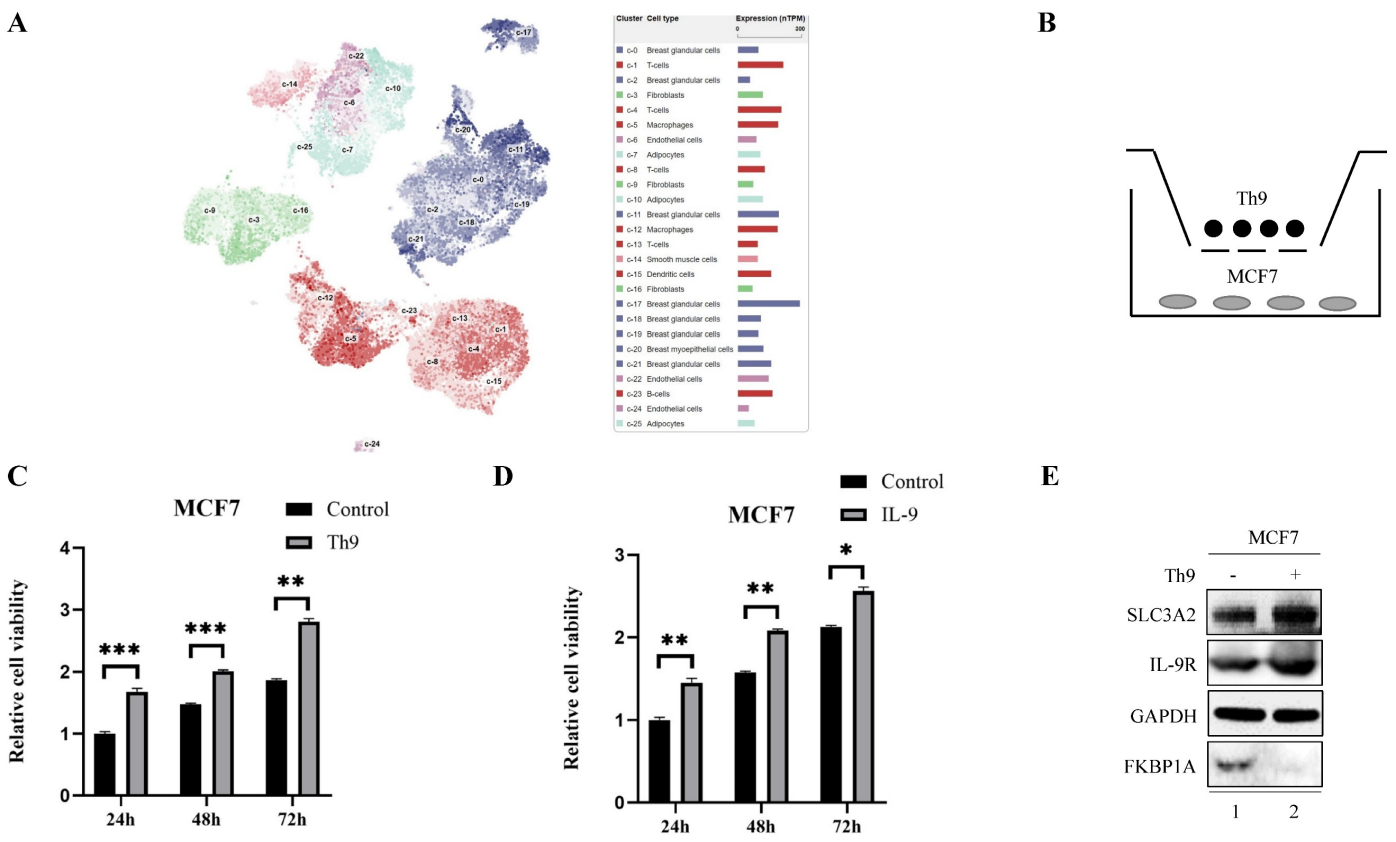

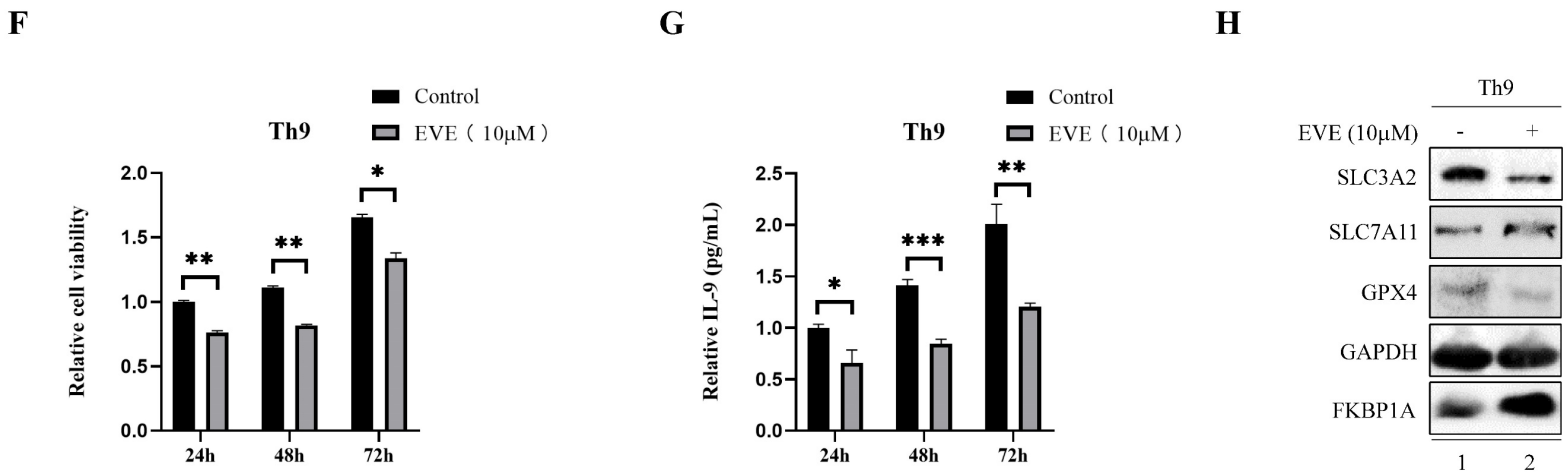
** Th9 cells enhance the breast cancer cells growth and everolimus inhibits proliferation of Th9 cells via FKBP1A/SLC3A2 axis. A** The clustering of SLC3A2 gene expressed in single cell types of the breast. **B** Co-culture model of Th9 cells on MCF7 cells. **C** MCF7 cells were treated with Th9 cells for 24, 48, or 72 h; cell viabilities were measured. **D** MCF7 cells were treated with IL-9 (100ng/mL) for 24, 48, or 72 h; cell viabilities were measured.** E** MCF7 cells were co-cultured with Th9 cells for 72 h. SLC3A2, IL-9R and FKBP1A protein expression were measured via Western blotting.** F** Th9 cells were treated with everolimus (10 uM) for 24, 48, or 72 h, and cell viability was assayed. **G** Th9 cells were treated with everolimus (10 uM) for 24, 48, or 72 h, and secreted IL-9 was measured by ELISA. **H** Th9 cells were treated with everolimus (10 uM) for 72 h. SLC3A2, SLC7A11, GPX4 and FKBP1A protein expression were measured via Western blotting. *p<0.05, **p<0.01, ***p<0.001.

Furthermore, we found a large proportion of T lymphocytes among single cell types of the breast via HPA database (Fig. 6A), Recently, Th9 cells, a subgroup of CD4^+^ T lymphocytes marked by the secretion of IL-9, have been reported involved in tumor progression 20, 21. To investigate the impact of Th9 cells on MCF7 cells mimicking BC microenvironment, we co-cultured two cells via transwell assay (Fig. 6B), the result showed Th9 cells enhanced the cell viability of MCF7 cells in time-dependent manner (Fig. 6C), implying that Th9 cells might act as an oncogenic factor in the TME of BC. Meanwhile, we also demonstrated that IL-9 enhanced the cell viability of MCF7 cells in time-dependent manner (Fig. 6D). Along with the findings reported by Sun et al. that BC patients with high IL-9 expression also tended to present high IL-9R expression^30^, we found co-cultured Th9 cells could upregulate IL-9R and SLC3A2 expression, but downregulate FKBP1A expression level in MCF7 cells (Fig. 6E), implying that Th9 cells might regulate the FKBP1A/SLC3A2 axis of BC cells via IL-9/IL-9R axis. Since our findings that everolimus could also effectively inhibit the proliferation of Th9 cells (Fig. 6F), as well as the IL-9 secretion of Th9 cells (Fig. 6G), we aimed to explore the FKBP1A/SLC3A2 axis in Th9 cells. Unsurprisingly, everolimus also effectively inhibited the expression of SLC3A2 and GPX4, but promoted the expression of FKBP1A in Th9 cells (Fig. 6H).

### Bioinformatic analysis of FKBP1A in breast cancer

**Figure 7:**
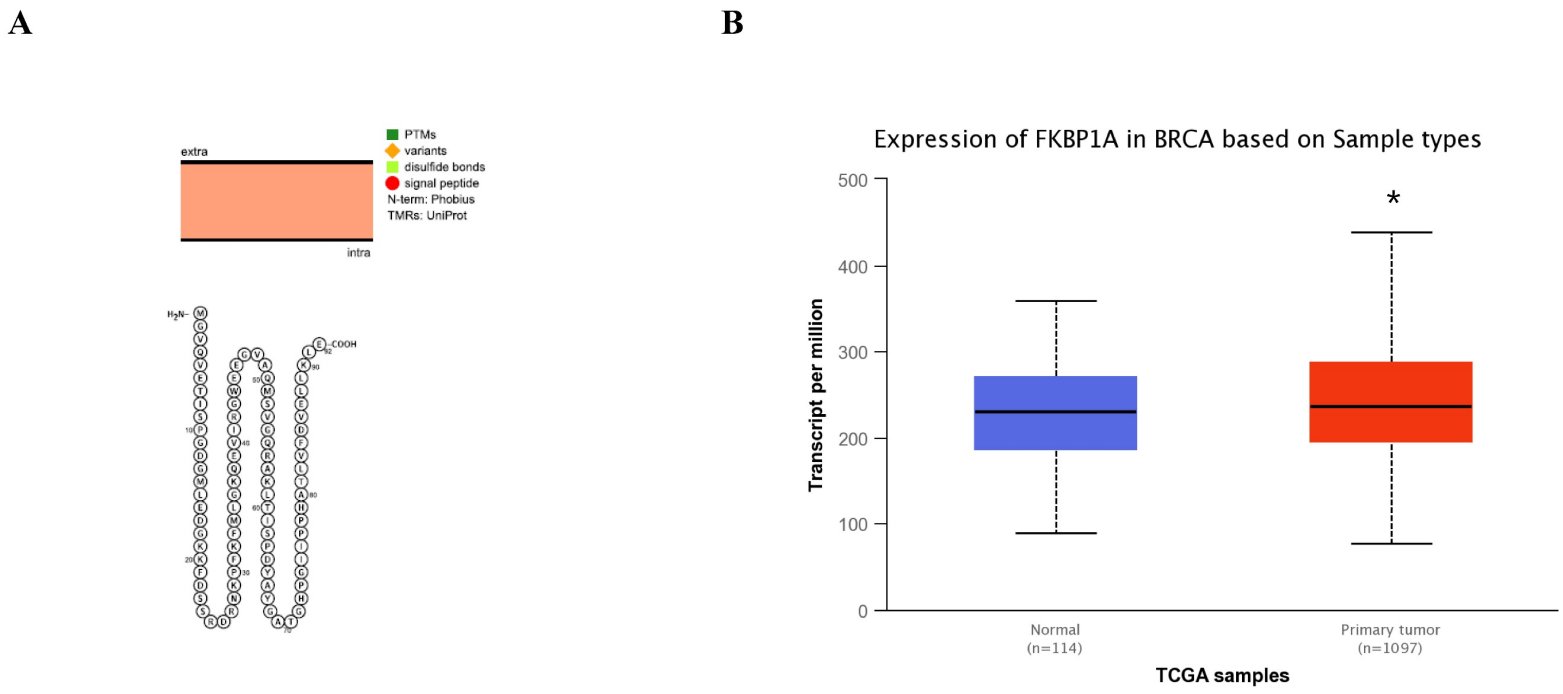

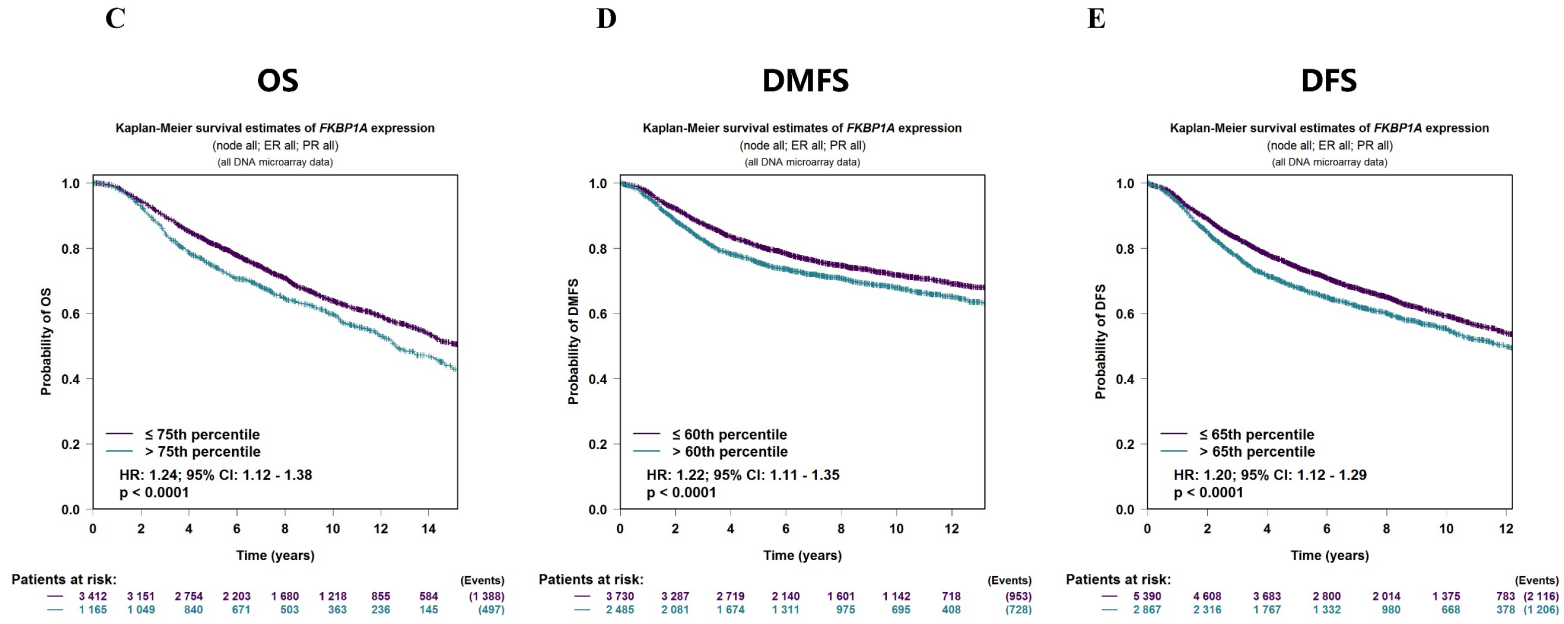

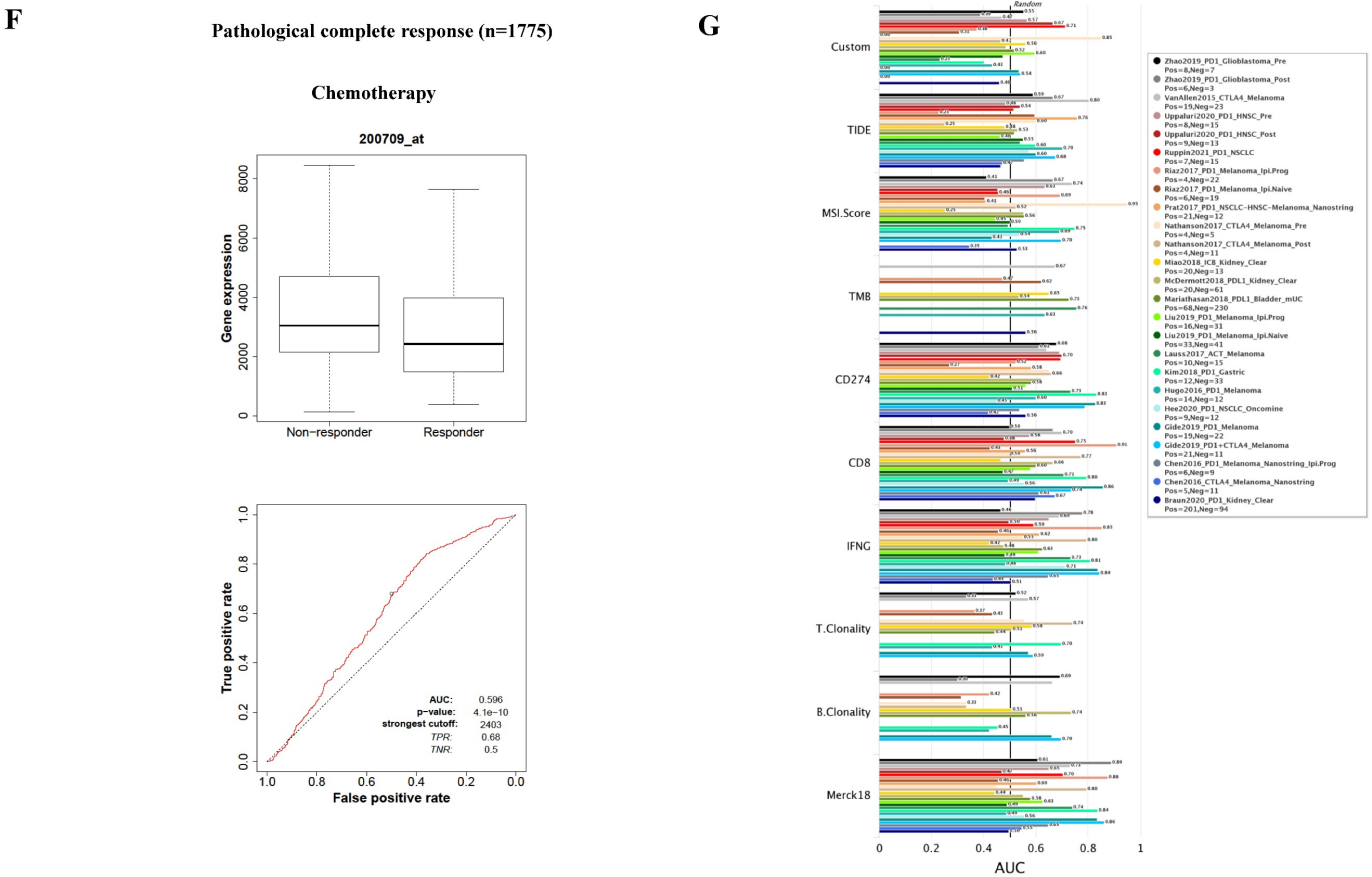

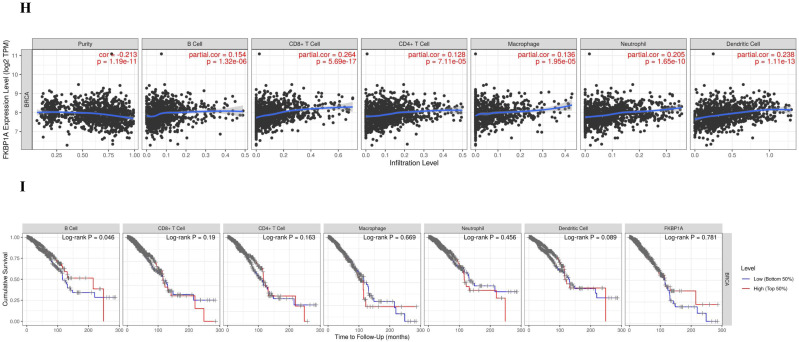

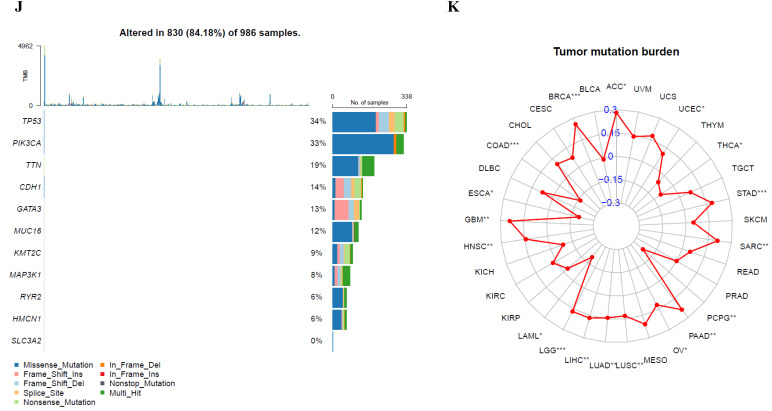
**Bioinformatic analysis of FKBP1A in breast cancer. A** FKBP1A protein topology showed the cytoplasm location. **B** The mRNA level of FKBP1A in breast cancer from UALCAN database. **C-E** Relationship of FKBP1A with overall survival, distant metastasis free survival and disease-free survival of breast cancer patients using bc-GenExMiner v4.7 database. **F** The receiver operating characteristic curve plot of the association between FKBP1A expression and responses to chemotherapy in breast cancer cohort. **G** Bar plot showing the biomarker relevance of FKBP1A compared to standardized cancer immune evasion biomarkers in immune checkpoint blockade sub-cohorts. The area under the receiver operating characteristic curve was applied to evaluate the predictive performances of the test biomarkers on the immune checkpoint blockade response status. **H** Spearman correlations between expression of FKBP1A and infiltration level of diverse immune cells in breast cancer using TIMER database. **I** Survival analysis of FKBP1A expression and diverse immune cells infiltration level in breast cancer. **J** Waterfall plot showing FKBP1A mutations compared with common mutated genes in TCGA database. **K** Radar map of connection of FKBP1A and tumor mutation burden in pan-cancer. *p<0.05, **p<0.01, ***p<0.001.

The FKBP1A protein was detected in cytoplasm (Fig. 7A), and UALCAN database revealed that FKBP1A was significantly upregulated in BC patients at mRNA level (Fig. 7B). Upregulated FKBP1A was associated with shorter OS, DMFS and DFS of BC patients (Fig. 7C-E). Further result showed that higher FKBP1A expression benefitted less from chemotherapy when pathological complete response was considered (Fig. 7F). Using TIDE database, we found that FKBP1A also had an area under the receiver operating characteristic curve of > 0.5 in 12 of the 25 immune checkpoint blockade sub-cohorts (Fig. 7G). Although we elucidated that FKBP1A was closely associated with numerous immune cell infiltrations (Fig. 7H), but it almost did not affect the role of these immune cells in patient prognosis (Fig. 7I). As for the relation of FKBP1A and genetic alterations in BC, we found that FKBP1A had little change compared with common mutated genes in TCGA database (Fig. 7J), but it contributed a top significant impact on BC tumor mutational burden (Fig. 7K).

## Discussion

SLC3A2 is a key chaperone for xCT in the process of ferroptosis, metabolism and proliferation in cancer cells 31-33. Owing to these diverse molecular functions, overexpression of SLC3A2 has been found associated with the development and progression of various types of cancer. For instance, Sun et al. demonstrated that SLC3A2 exerted a malignant effect via increasing proliferation and decreasing apoptosis in oral cancer patients 33. Although some studies have reported the relationship between SLC3A2 and BC 8, 34, a comprehensive and systematic analysis is still lacking. Our study firstly illustrated the relationships of expression levels, genetic or epigenetic alterations of SLC3A2 with prognoses, drug response, clinicopathological characteristics, immune cell infiltration, enrichment analysis, and single cell sequencing in BC using online databases.

It is well-known that TME is consist of cellular and non-cellular elements, providing nutrition support for the growth of tumor cells, helping tumor cells against the immune killing and immunosuppression including CTL 35. Our results showed that SLC3A2 was only remarkably negatively correlated with CTL in BC TME, and only in BC patients with low SLC3A2 expression, the higher expression of CTL was positively correlated with patients' OS. BC has been reported characterized by an increase in metabolism, while immune cells remain at a disadvantage in the nutrient deficient TME 36. Among different 458 members of the solute carrier membrane-bound nutrient transporters, SLC3A2 plays a major role in promoting uptake procedure of glutamine and leucine 37. Since we found SLC3A2 was negatively associated with numerous immune cell infiltrations, further mining the molecular regulation of SLC3A2 will provide insight into novel strategies to activate immune cells in the TME of BC.

Human FK506-binding protein family includes 18 FKBPs up to date, which could target on diverse pathways in cardiac function, embryonic development, stress response and cancer tumorigenesis 38. Our previous work has demonstrated that FKBP4 regulates NRF2 via regulating nuclear translocation of NR3C1 in BC 39. Xu et al. identified that PHB1 specifically interacted with FKBP8 through immunoprecipitation-mass spectrometry-based proteomic analysis 40. For the first time, we found that FKBP1A could bind with SLC3A2, and negatively regulated SLC3A2 expression during the processes of everolimus inducing ferroptosis of BC cells and promoting anti-proliferation of Th9 lymphocytes.

Currently, there is still lack of regulation mechanisms between FKBP1A/SLC3A2 and Th9 cells, and only one study suggests that FKBP5 is solely correlated with CD4^+^ T-lymphocytes polarization 41. In our study, we firstly demonstrated that everolimus inhibited the proliferation of Th9 cells through FKBP1A/SLC3A2 axis, but this mechanism is not clearly articulated due to low plasmid and siRNA transfection efficiency of Th9 cells. Therefore, subsequent studies on FKBP1A/SLC3A2 axis need to focus on the exosomal communication between tumor cells and Th9 cells in BC TME.

Numerous evidence has demonstrated that immunotherapy plays an important role in BC, and immunotherapy could alter or enhance the immune system when combined with everolimus, *e.g.*, IL-15 gene therapy combined everolimus was reported useful for the treatment of metastatic BC 42. Here we implied that everolimus could regulate Th9 cells proliferation to affect BC cells viability when co-culture two kinds of cells, combination therapy to target FKBP1A/SLC3A2 axis should be considered to maximise the effectiveness of immunotherapy in this setting. With the development of immunotherapies including immune checkpoint inhibitors and CAR-T therapy 43, along with Th9-targeting methods might lead to better prognoses in BC patients.

## Materials and Methods

### Cell culture

MCF7, T47D, Th9 and 293T cells were obtained from the American Type Culture Collection (ATCC). MCF7 and 293T were cultured in Dulbecco's modified Eagle's medium. T47D and Th9 were cultured in Roswell Park Memorial Institute (RPMI) medium. Growth media were supplemented with 10% fetal calf serum and penicillin/streptomycin. All human cell lines were cultured at 37 °C in a humidified incubator supplied with 5% CO_2_.

### Antibodies and reagents

Antibodies were used in the following dilutions: SLC3A2 (1:1000, Affinity, #DF7468), FKBP1A (1:1000, Proteintech, #10273-1-AP), SLC7A11 (Abcam, ab37185), GPX4 (Abcam, ab125066), IL-9R (Santa Cruz, sc-515622), GAPDH (1:1000, Proteintech, #60004-1-Ig), HA (1:1000, Proteintech, #51064-2-AP), Flag (1:1000, Sigma, #F3165). Secondary antibody goat anti-mouse (1:2500, HuaBio, #HA1006), secondary antibody goat anti-rabbit (1:2500, HuaBio, #HA1001). Everolimus was purchased from APExBIO (#A8169), and resolved in DMSO at 10mM. Erastin was purchased from meilunbio (#MB4114), and resolved in DMSO at 10mM. IL-9 was purchased from MCE (#HY-P7046), and resolved in PBS at 100μg/mL.

### Western blotting

Knockdown and overexpression efficiencies and biochemical responses were analyzed by western blotting. Cells were lysed in RIPA lysis buffer (EMD Millipore Corp.). Separated proteins were transferred to PVDF membranes and blocked in 5% milk in Tris-buffered saline, with 0.05% Tween-20. Immunodetection was done with various primary antibodies. Appropriate horseradish peroxidase-conjugated secondary antibodies were used and signals were visualized with Bio-Rad chemiluminescence by Bio-Rad ChemiDoc™ MP Imaging System.

### Co-IP

Cells for co-IP were lysed in lysis buffer (50 mM Tris, pH 7.5, with 150 mM NaCl, 0.5% NP-40, and protease inhibitor and phosphatase inhibitor cocktail tablets (Roche) at 4 °C for 30 min. After sonication and centrifugation, cell lysates were incubated with HA beads (Sigma) at 4 °C overnight on a rotator. After six washes with wash buffer (20 mM Tris, pH 7.5, 100 mM NaCl, 0.05% Tween-20, 0.1 mM EDTA), 50 μL of elution buffer (100 mM glycine-HCl, pH 2.5) was added to resuspend the beads, and the eluted proteins were obtained by centrifugation, followed by SDS-PAGE and immunoblotting analysis.

### Plasmids

FKBP1A-HA, SLC3A2-Flag and the control plasmid were constructed by Tsingke Biotechnology Co., Ltd.

### Transient transfection

MCF7, T47D or 293T cells cultured in 12-well tissue culture plates were transiently transfected with plasmids using Lipofectamine^®^ 2000 Reagent (Invitrogen) or siRNAs using Lipofectamine^®^ RNAiMAX Reagent (Invitrogen) as instructed by the manufacturer. The siRNA targeting SLC3A2 or FKBP1A, as well as negative control siRNA were purchased from RIBBIO (Guangzhou, China). 72 h later, the whole-cell extract was prepared for western blot analysis.

### Gene Silence

The shRNA against SLC3A2 was purchased from RIBBIO (Guangzhou, China). The shRNA targeting sequences of SLC3A2 were as follows: CCGGCAATTCACAAGAACCAGAAGGCTCGAGTTCTGGTTCTTGTGAATTGGATTTTT. In brief, 293T packaging cells were transfected with 800 ng shRNA in combination with the packaging plasmids 200 ng lenti-VSV-G, 400 ng lenti-RRE, and 140 ng lenti-REV. Virus containing supernatant was harvested at 48 h after transfection and filtered through a 0.45 mM syringe filter with the addition of 10 mM DEAE. Supernatants were used to infect target cells in another 12 h period.

### Cell proliferation assay

Cell proliferation was analyzed using a Cell Counting Kit-8 (CCK-8) (DOJINDO). All cells were seeded into 96-well plates at a density of 5000 cells/well in a 100 µl volume and incubated at 37°C under 5% CO_2_ for 24, 48, 72 h, followed by the addition of 10 μl of CCK-8 solution. The absorbance in each well was measured after 1 h incubation using a microculture plate reader at a test wavelength of 450 nm. Three replicate wells were set up in each group, and three independent experiments were performed.

### Edu Staining

The Edu-488 cell proliferation detection kit was used for cell proliferation detection, in which the thymidine analog Edu was incorporated and labeled in the process of DNA synthesis. 100 μL of Edu working solution (10 μM) was added into MCF7 or T47D cells in the 96-well plate for incubation for 2 h. Subsequently, the cells were fixed in 4% paraformaldehyde for 15 min, sealed in PBS solution containing 3% bovine serum albumin, permeabilized in 0.3% Triton X-100 solution for 15 min, and washed with PBS (5 min/time). Finally, the staining was observed at the wavelength of 495 nm under a microscope.

### Co-cultures

MCF7 cells were co-cultured with Th9 cells by using a Transwell culture system with 0.4 μm pore polyester membrane inserts (Corning HTS Transwell, Corning, NY, USA) for 3 days. Specifically, MCF7 cells were seeded on plates, while Th9 cells were seeded in Transwell chambers.

### Total ROS assay

The total ROS level in cells was assessed using a Reactive oxygen species detection kit (Beyotime, S0033S). Cells were treated as indicated, and then, 10 μmol/L DCFH-DA diluted in serum-free medium was added and incubated with the cells at 37 °C for 20 min. Excess DCFH-DA was removed by washing the cells three times with serum-free cell culture medium. DCFH-DA can be hydrolyzed by intracellular esterases to produce DCFH. Labeled cells were trypsinized and resuspended in serum-free medium. Intracellular reactive oxygen species can oxidize nonfluorescent DCFH to produce DCF, which is fluorescent. The fluorescence of DCF was analyzed using flow cytometry.

### GSH assay

The relative GSH concentration in cell lysates was assessed using a total Glutathione Assay Kit (Beyotime, S0052) according to the manufacturer's instructions. Briefly, Glutathione reductase reduces oxidized glutathione (GSSG) to reduced glutathione (GSH), which reacts with the chromatin substrate DTNB to produce yellow TNB and GSSG. The combination of the two reactions reveals the total glutathione (GSSG + GSH), and the amount of TNB (yellow) formation represents the amount of total glutathione. Thus, the total glutathione content can be calculated by measuring A412.

### IL-9 ELISA

After incubation for 72 h under 37 °C der 5% CO_2_ with/without everolimus, the supernatant and the cells were separated via 5-minute centrifugation at 1000 g. IL-9 ELISA was performed on the supernatant using Human IL-9 ELISA Kit (CUSABIO).

### *In Vivo* Tumor Xenograft Assays

Four-week-old female BALB/c nude mice were purchased from Vital River Laboratory Animal Technology Co, Ltd (Beijing, P.R. China). Animal experimental procedures were approved by the Medical Ethics Committee of Zhejiang Provincial People's Hospital. The four-week-old female mice were randomized into different groups. T47D shControl and T47D shSLC3A2 cells (1 ×10^6^ cells/mice) were implanted subcutaneously into the left underarm of nude mice. After tumor formation, everolimus (5 mg/kg) was administered orally to its respective animal treatment groups for 8 days. Tumor volume (mm^3^) was measured every two days and calculated by the formula (length × width × width)/2. After the tumors were surgically removed, a portion of the tumors was immediately fixed in 10% buffered formalin for immunohistochemistry.

### Bioinformatics analysis

The localization and expression of SLC3A2 and FKBP1A protein were generated by PROTTER database 44 and HPA database 45. In PROTTER, we used “topology” section to analyse the structure of SLC3A2 and FKBP1A, in HPA, we used “subcellular” section to analyse the subcellular distribution of SLC3A2 and FKBP1A. The immunofluorescence of the subcellular distribution of SLC3A2 protein were generated by from the HPA database. The expression of SLC3A2 and FKBP1A mRNA were generated by TIMER2 database 46 and UALCAN database 47 according to the database instruction. Disease network interaction analysis of SLC3A2 was generated by OPENTARGET database 48. Prognostic merits of FKBP1A was generated by the “prognostic” section of bc-GenExMiner v4.7 database 49. ROC curve plot of the association between SLC3A2 and FKBP1A expression and pathological complete response or relapse-free survival at 5 years were generated by GSCALite database 50. The clinical traits of SLC3A2 in BC were generated by the “expression” section of UALCAN database. The immunological traits of SLC3A2 and FKBP1A in BC were generated by immunological related section of TIDE database 51, TIMER database 52 and TISIDB database 53. Genetic alterations and co-occurrence analyses of SLC3A2 were generated by the “mutation distribution” section of COSMIC database 54 and the “mutations” section of cbioportal database 55. The mutation frequency of SLC3A2 and FKBP1A were obtained using “waterfalls” R package. The radar plots for TMB of SLC3A2 and FKBP1A were obtained using “Fmsb” R package. The methylation traits of SLC3A2 were generated by the “methylation” section of UALCAN database and TIDE database. Single cell analysis of SLC3A2 in BC or immune cells were generated by the “functional relevance in distinct cell groups” section of CancerSEA database 56 and HPA database.

### Statistics

Two-tailed student's t-test was used in this study. Data shown was mean±SD from at least three independent experiments. Statistical probability was expressed as *p < 0.05, **p < 0.01, ***p < 0.001. p values generated by different databases were automatically calculated by each database.

## Supplementary Material

Supplementary figures.
