# Supplementary material for: Integrated analysis of FKBP1A/SLC3A2 axis in everolimus inducing ferroptosis of breast cancer and anti-proliferation of T lymphocyte
Source: Int J Med Sci. 2023 Jun 26;20(8):1060–78. doi: 10.7150/ijms.84872 (PMC10357440; doi:10.7150/ijms.84872)

Supplementary description

Figure S1 Relation of SLC3A2 and therapeutic responses in breast cancer. **A-C** The receiver operating characteristic curve plot of the association between SLC3A2 expression and responses to endocrine therapy, anti-HER2 therapy, and chemotherapy in breast cancer cohort considering pathological complete response. **D-F** The receiver operating characteristic curve plot of the association between SLC3A2 expression and responses to endocrine therapy, anti-HER2 therapy, and chemotherapy in breast cancer cohort considering relapse-free survival at 5 years.

Figure S2 Relation of SLC3A2 and clinicopathological characteristics in breast cancer. **A-H** Correlations between SLC3A2 mRNA expression and genders, cancer stages, nodal metastasis status, molecular subtypes, races, ages, menopause status and TP53 mutation status. **p<0.01, ***p<0.001.

Figure S3 Relation of SLC3A2 and single cell sequencing in breast cancer. **A** Correlations between SLC3A2 and diverse functional states in breast cancer from CancerSEA database. **B-I** Box diagram and scatter diagram showing the SLC3A2 expression among four single cell datasets (EXP0052, EXP0053, EXP0054, EXP0055).

Figure S4 Everolimus inhibits the proliferation of T47D cells. **A** T47D cells were treated with everolimus (10 uM) for 0, 24, 48, or 72 h, and cell viability was assayed. **B** Comparison of T47D cell proliferative capacity among control and everolimus (10 uM) groups at 72 h via Edu staining. Scale bar = 50 um. **C** T47D cells were treated with varying doses of everolimus alone or in combination with different doses of erastin for 48 h; cell viabilities were measured. *p<0.05, **p<0.01, ***p<0.001.

Figure S5 SLC3A2 influences cell viability of breast cancer cells by everolimus treatment. **A** T47D cells were treated with everolimus (10 uM) for 0, 24, 48, or 72 h with or without the overexpression of SLC3A2. Cell viability was assayed. **B** MCF7 cells were treated with everolimus (10 uM) for 0, 24, 48, or 72 h with or without the downexpression of SLC3A2. Cell viability was assayed. **C** T47D cells were treated with everolimus (10 uM) for 0, 24, 48, or 72 h with or without the downexpression of SLC3A2. Cell viability was assayed. *p<0.05.

Figure S1

Pathological complete response (n=1775)

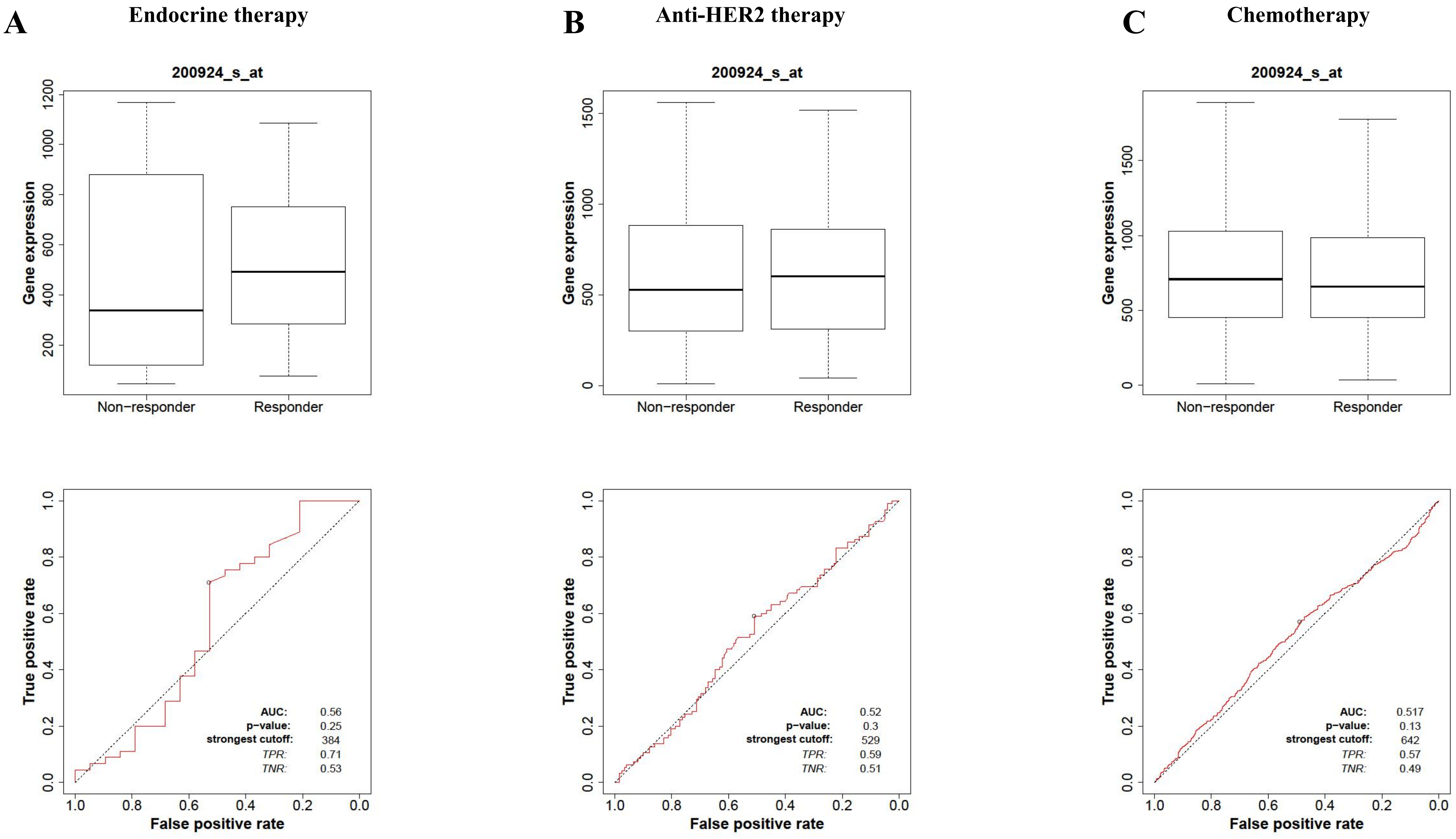

Relapse-free survival at 5 years (n=1329)

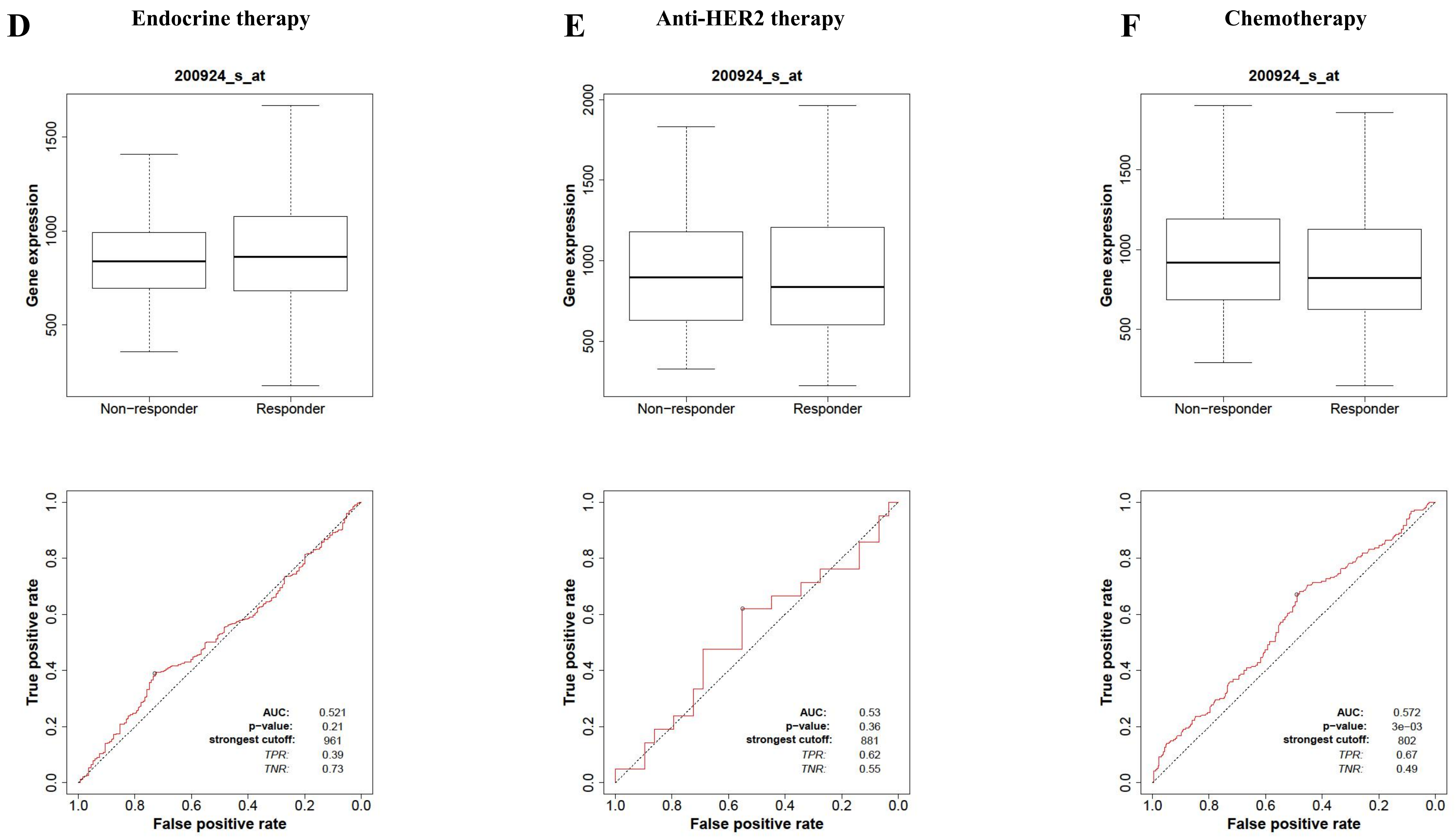

Figure S2

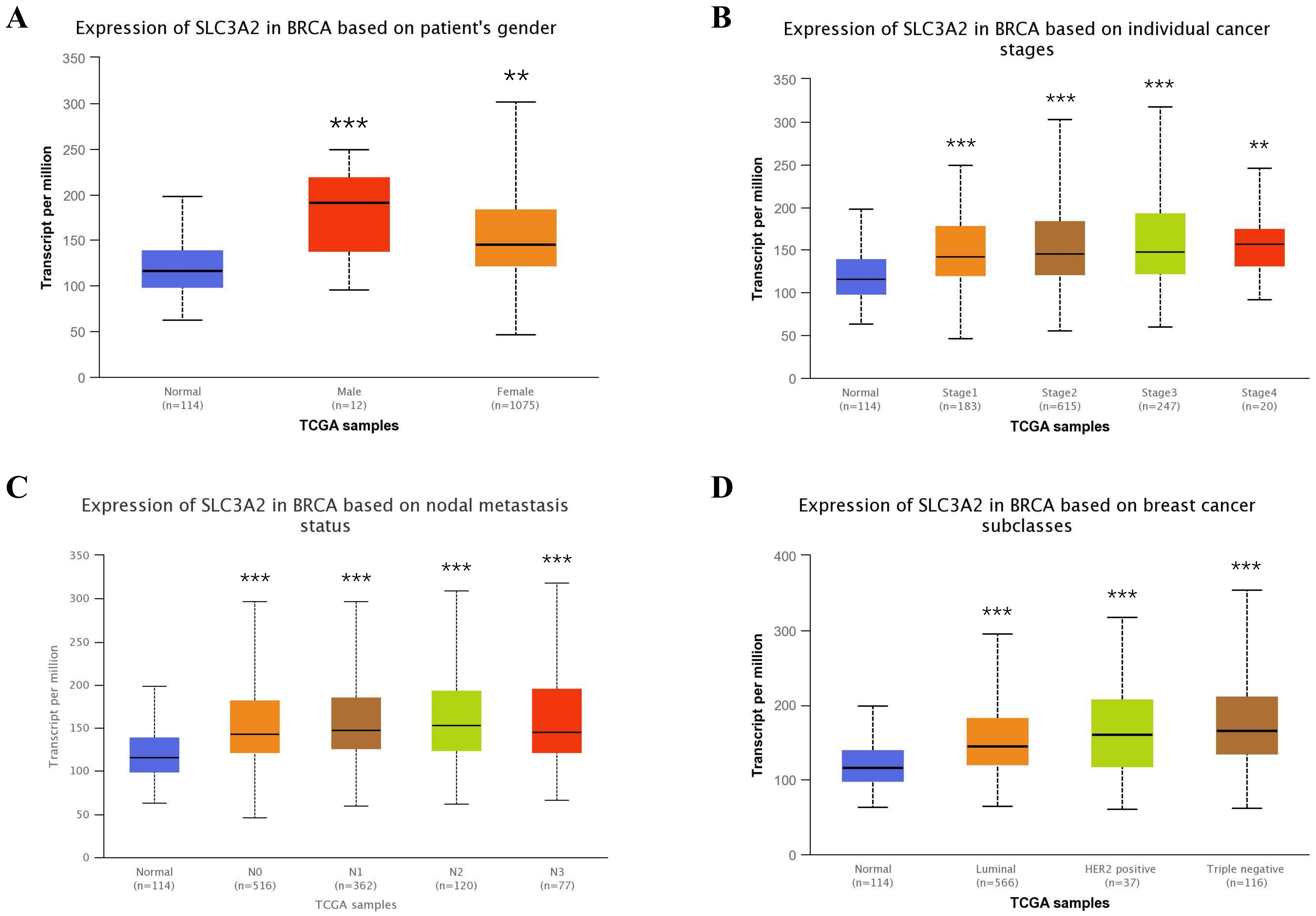
A
Expression of SLC3A2 in BRCA based on patient's gender
Transcript per million
350
300
250
200
150
100
50
0
***
**
Normal
(n=114)
Male
(n=12)
Female
(n=1075)
TCGA samples
B
Expression of SLC3A2 in BRCA based on individual cancer stages
Transcript per million
350
300
250
200
150
100
50
0
***
***
***
**
Normal
(n=114)
Stage1
(n=183)
Stage2
(n=615)
Stage3
(n=247)
Stage4
(n=20)
TCGA samples
C
Expression of SLC3A2 in BRCA based on nodal metastasis status
Transcript per million
350
300
250
200
150
100
50
0
***
***
***
***
Normal
(n=114)
N0
(n=516)
N1
(n=362)
N2
(n=120)
N3
(n=77)
TCGA samples
D
Expression of SLC3A2 in BRCA based on breast cancer subclasses
Transcript per million
400
300
200
100
0
***
***
***
Normal
(n=114)
Luminal
(n=566)
HER2 positive
(n=37)
Triple negative
(n=116)
TCGA samples

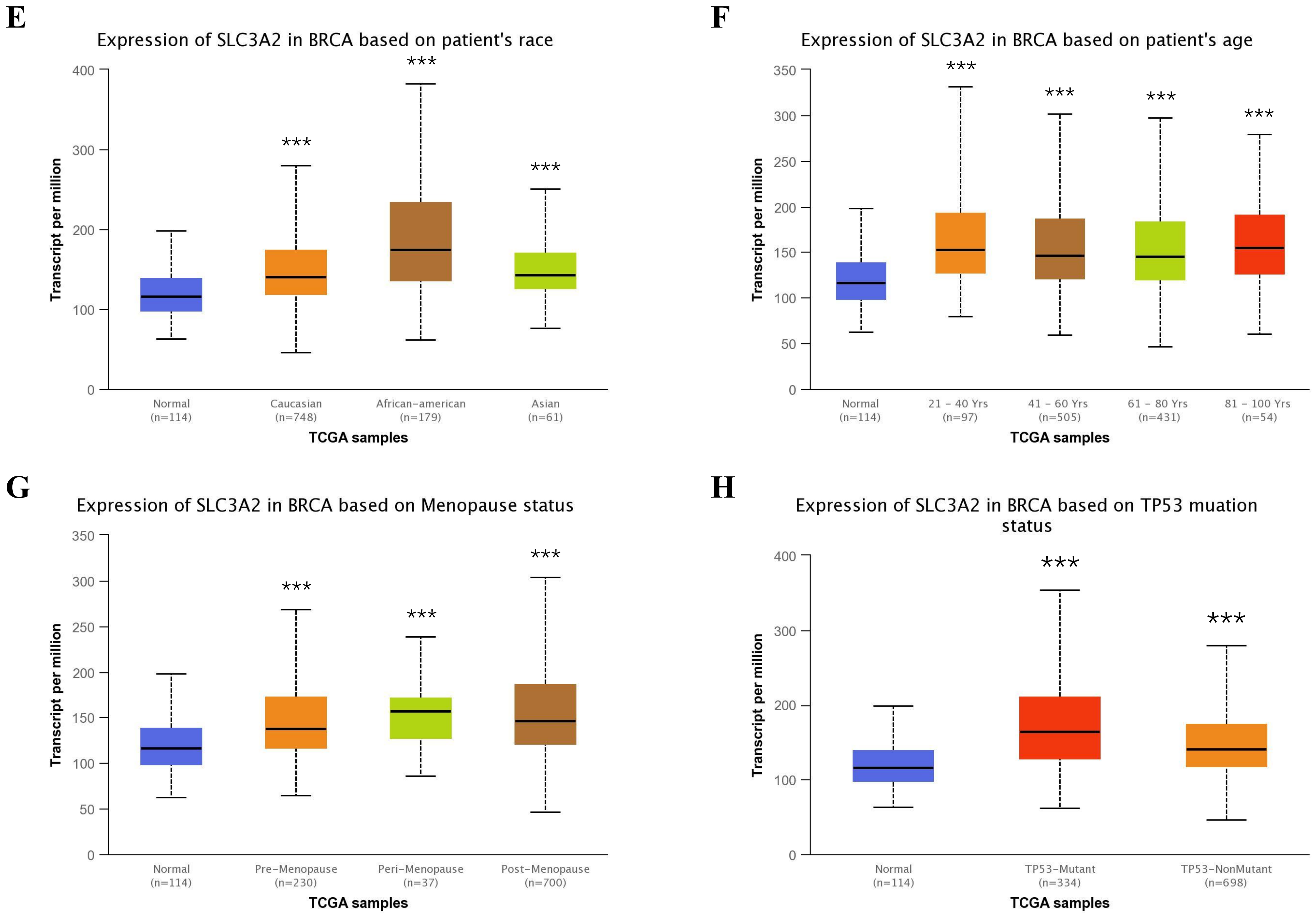
E
Expression of SLC3A2 in BRCA based on patient's race
Transcript per million
0
100
200
300
400
***
***
***
Normal (n=114)
Caucasian (n=748)
African-american (n=179)
Asian (n=61)
TCGA samples
F
Expression of SLC3A2 in BRCA based on patient's age
Transcript per million
0
50
100
150
200
250
300
350
***
***
***
***
Normal (n=114)
21 - 40 Yrs (n=97)
41 - 60 Yrs (n=505)
61 - 80 Yrs (n=431)
81 - 100 Yrs (n=54)
TCGA samples
G
Expression of SLC3A2 in BRCA based on Menopause status
Transcript per million
0
50
100
150
200
250
300
350
***
***
***
Normal (n=114)
Pre-Menopause (n=230)
Peri-Menopause (n=37)
Post-Menopause (n=700)
TCGA samples
H
Expression of SLC3A2 in BRCA based on TP53 muation status
Transcript per million
0
100
200
300
400
***
***
Normal (n=114)
TP53-Mutant (n=334)
TP53-NonMutant (n=698)
TCGA samples

Figure S3

A

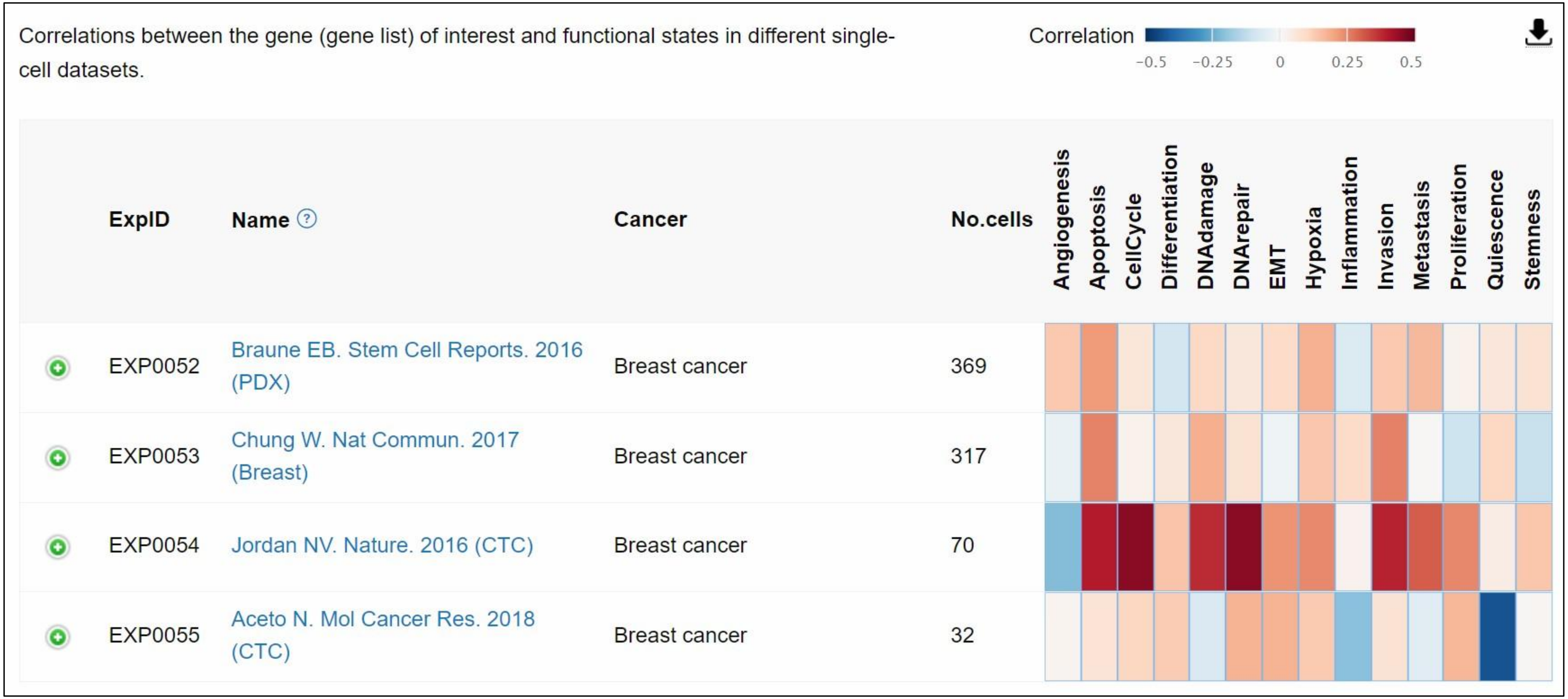

ExpID	Name	Cancer	No.cells
EXP0052	Braune EB. Stem Cell Reports. 2016 (PDX)	Breast cancer	369
EXP0053	Chung W. Nat Commun. 2017 (Breast)	Breast cancer	317
EXP0054	Jordan NV. Nature. 2016 (CTC)	Breast cancer	70
EXP0055	Aceto N. Mol Cancer Res. 2018 (CTC)	Breast cancer	32

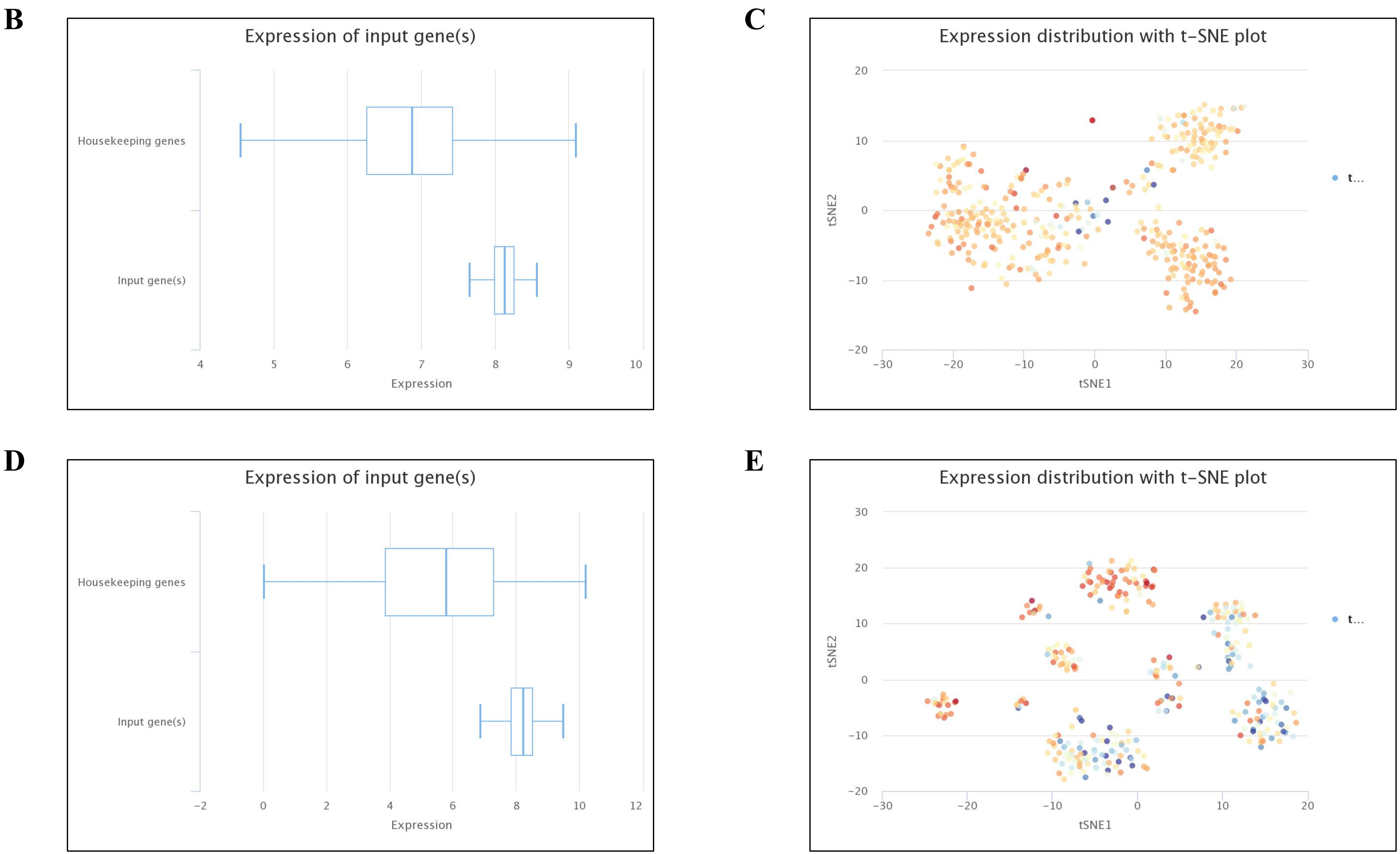
B
Expression of input gene(s)
Housekeeping genes
Input gene(s)
4 5 6 7 8 9 10
Expression
C
Expression distribution with t-SNE plot
20 10 0 −10 −20
tSNE2
−30 −20 −10 0 10 20 30
tSNE1
t...
D
Expression of input gene(s)
Housekeeping genes
Input gene(s)
−2 0 2 4 6 8 10 12
Expression
E
Expression distribution with t-SNE plot
30 20 10 0 −10 −20
tSNE2
−30 −20 −10 0 10 20
tSNE1
t...

F

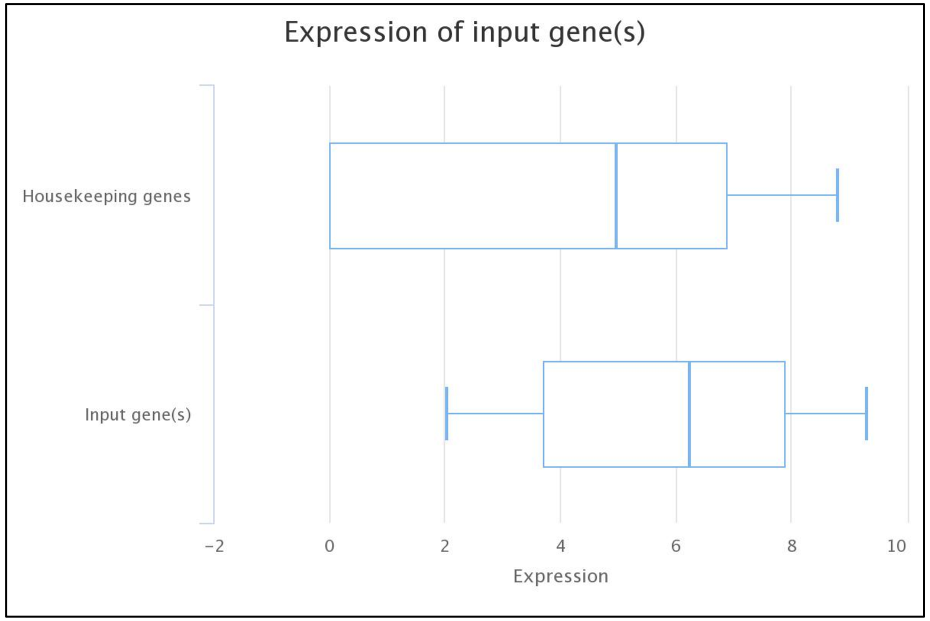
Expression of input gene(s)
Housekeeping genes
Input gene(s)
−2 0 2 4 6 8 10
Expression

G

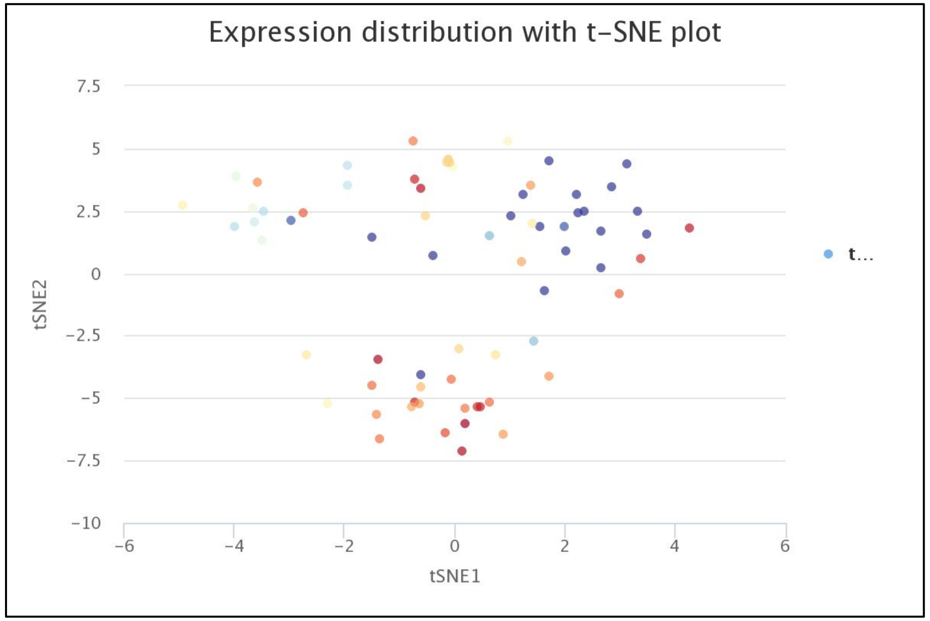
Expression distribution with t-SNE plot
7.5 5 2.5 0 −2.5 −5 −7.5 −10
tSNE2
−6 −4 −2 0 2 4 6
tSNE1
t...

H

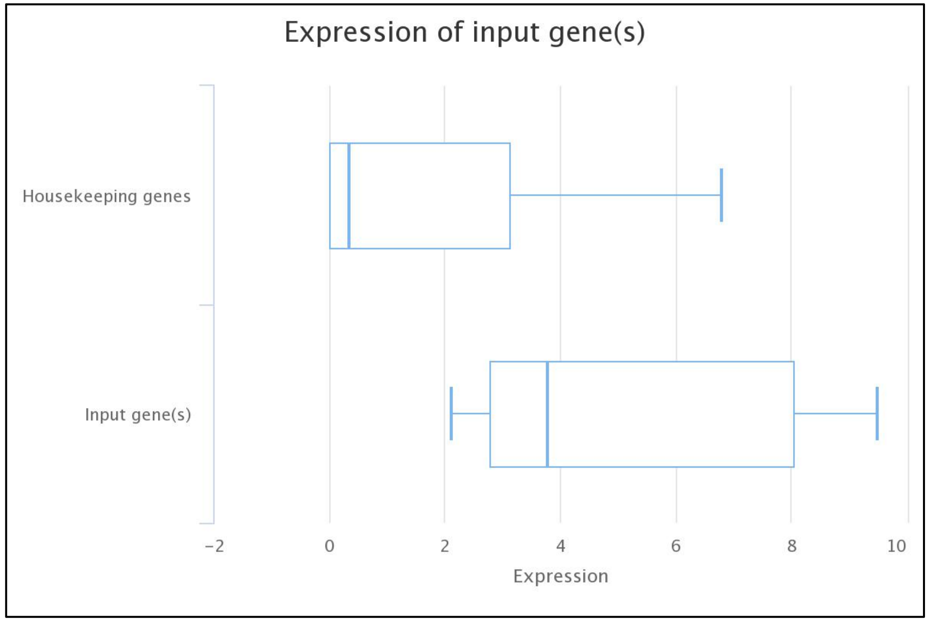
Expression of input gene(s)
Housekeeping genes
Input gene(s)
−2 0 2 4 6 8 10
Expression

I

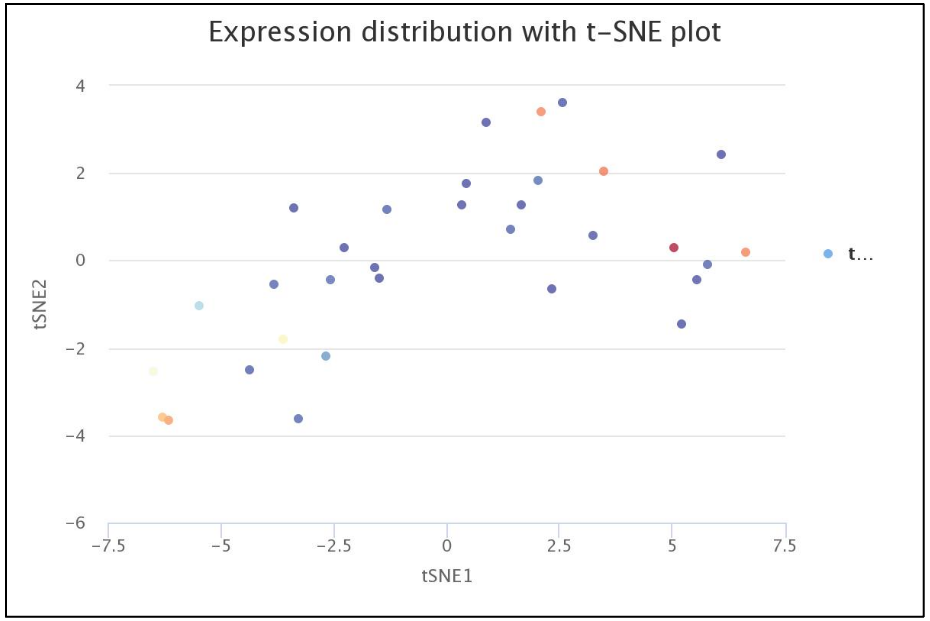
Expression distribution with t-SNE plot
4 2 0 −2 −4 −6
tSNE2
−7.5 −5 −2.5 0 2.5 5 7.5
tSNE1
t...

Figure S4

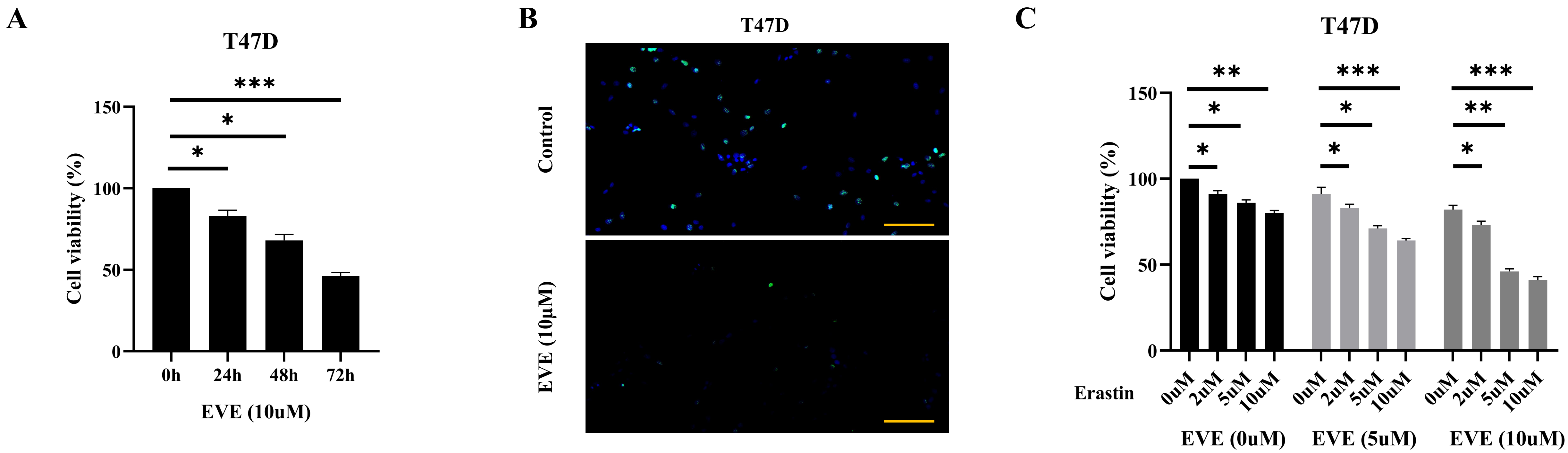
A
T47D
***
*
*
150
100
50
0
Cell viability (%)
0h
24h
48h
72h
EVE (10uM)
B
T47D
Control
EVE (10μM)
C
T47D
**
***
***
*
*
**
*
*
*
150
100
50
0
Cell viability (%)
Erastin
0uM
2uM
5uM
10uM
EVE (0uM)
EVE (5uM)
EVE (10uM)

Figure S5

A

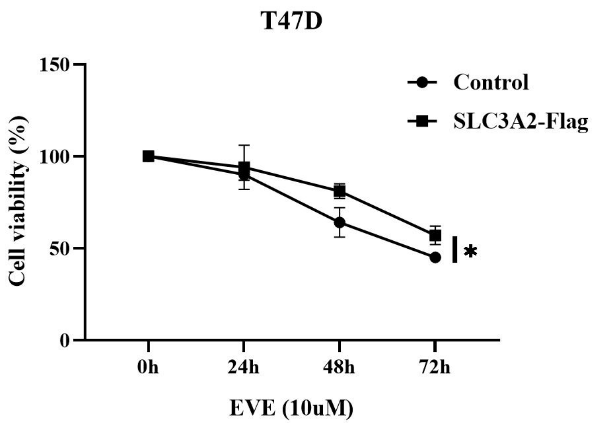

B

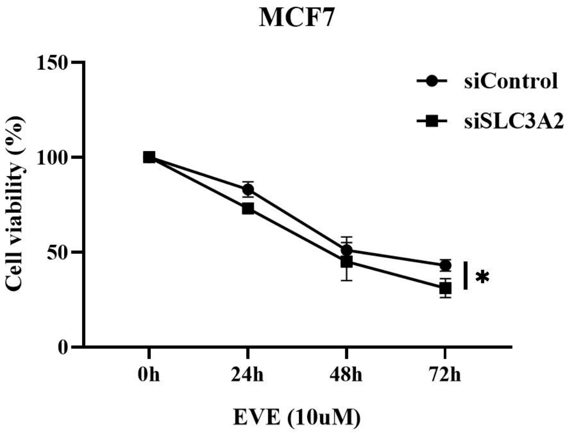

C

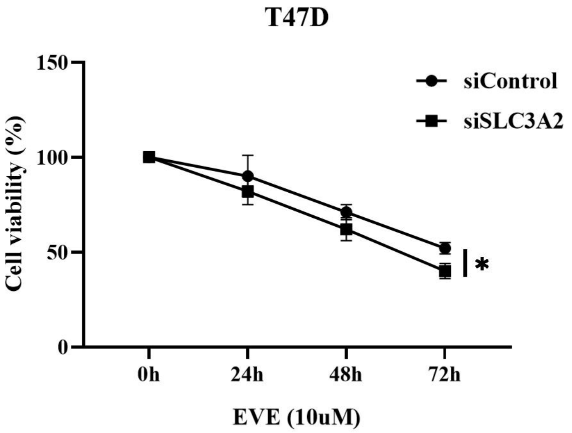

Supplement: Supplementary file 1 — Supplementary figures. [file ijmsv20p1060s1.pdf]
